# Isolation of a novel manganese-oxidizing bacterium *Lysinibacillus xylanilyticus* M125: characterization, structural evolution, and Cd-adsorption activity of biogenic Mn oxides produced by the strain

**DOI:** 10.3389/fmicb.2025.1622784

**Published:** 2025-08-21

**Authors:** Xiaoju Li, Xinyi Yuan, Yuxia Wei, Lianqi He, Yuanyuan Li, Meiquan Qiu, Yang Liu, Nannan Dong, Chengjia Zhang, Xin Pang

**Affiliations:** ^1^State Key Laboratory of Microbial Technology, Shandong University, Qingdao, China; ^2^School of Life Sciences, Shandong University, Qingdao, China; ^3^Binzhou Institute of Technology, Weiqiao-UCAS Science and Technology Park, Binzhou, China; ^4^Gansu Institute of Shandong University, Lanzhou, China

**Keywords:** *Lysinibacillus xylanilyticus*, manganese-oxidizing bacteria, biogenic manganese oxides, structural analysis, cadmium adsorption

## Abstract

**Introduction:**

Manganese-oxidizing bacteria (MOB) play a critical role in converting soluble Mn(II) to insoluble Mn(III/IV) oxides, which have been widely applied for environmental remediation, particularly in heavy metal pollution control. Therefore, the discovery of novel MOB strains is of great significance for advancing pollution mitigation and ecosystem restoration.

**Methods:**

In this study, a manganese-oxidizing bacterial strain was isolated from Mn-contaminated soil near an electroplating factory using selective LB medium supplemented with 10 mmol/L manganese chloride (MnCl_2_), and the Leucoberbelin Blue (LBB) assay was employed to screen and identify strains with strong Mn(II)-oxidation ability. The isolated strain was identified based on colony morphology, Gram staining, cellular morphology, physio-biochemical analysis, 16S rRNA sequencing, and phylogenetic analysis. The Mn-oxidation ability of this strain was determined by the LBB method. The effects of different pH, temperature, and Mn^2+^ concentrations on bacterial growth and Mn^2+^ oxidation were evaluated by OD_600nm_ and LBB method. The biogenic manganese oxides (BioMnO_x_) produced by strain M125 were characterized using TEM, XRD, XPS, and FTIR analyses. The cadmium adsorption capacity of BioMnO_x_ was assessed using inductively coupled plasma mass spectrometry.

**Results:**

A novel manganese-oxidizing bacterial strain was isolated from Mn-contaminated soil near an electroplating factory and identified as *Lysinibacillus xylanilyticus* M125. Evaluation of the influence of different pH, temperature, and Mn^2+^ concentrations on the growth of strain M125 showed that it grew well within a pH range of 5.0–10.0 and a temperature range of 15°C–40°C. It can tolerate Mn^2+^ concentrations up to 60 mM, indicating strong environmental resilience and potential for practical application. The manganese-oxidizing capacity of strain M125 was significantly affected by both Mn^2+^ concentration and pH. The oxidation activity increased with Mn^2+^ concentration up to 12 mM but declined at higher concentrations. Additionally, the strain demonstrated enhanced Mn-oxidation capability under higher pH conditions. BioMnO_x_, the product of strain M125 oxidation of manganese, had a relatively complex structure, containing a mixture of amorphous MnO_2_ and crystalline Mn_3_O_4_ phase. BioMnO_x_ exhibited various morphologies, including nanosheets, globular structures encased in sheaths, and extracellularly dispersed forms. Long-term cultivation further elucidated the morphological evolution of these oxides. Given the high surface area and porous nature of BioMnO_x_, its capacity for cadmium adsorption was also assessed. Over 99.5% of cadmium ions in water are adsorbed and removed by strain M125, highlighting its potential for cadmium pollution remediation.

**Discussion:**

Overall, this work introduces a new bacterial resource for Mn and Cd bioremediation and offers detailed insights into the structural and functional characteristics of BioMnO_x_, supporting its application in environmental biotechnology.

## 1 Introduction

With rapid industrialization, manganese contamination in soil has become increasingly serious, particularly due to its widespread use in smelting and electroplating industries. Therefore, the effective remediation strategies for manganese polluted soil are urgently needed. Among available approaches, biological oxidation method stands out for its operational simplicity, high efficiency, and environmentally friendly conditions (Su et al., 2014), revealing it a promising method with significant practical potential (Zhou et al., 2015; Sun et al., 2021; Wang Y. et al., 2022; He et al., 2019). Especially, the Mn-oxidizing bacteria have demonstrated strong remediation capabilities of manganese pollution. To date, a wide variety of Mn-oxidizing bacteria from oceans to soils have been discovered by researchers (Wang et al., 2016; Li et al., 2020; Zhang et al., 2019).

Mn-oxidizing bacteria can transform Mn(II) ions into Mn(III/IV) oxides. While Mn(II) oxidation by molecular oxygen is thermodynamically favorable, the reaction proceeds slowly without a catalytic agent. Mn-oxidizing bacteria can significantly accelerate this process through biological oxidation.

Biogenic manganese oxides (BioMnO_x_), which are the product of Mn-oxidizing bacteria, have attracted considerable attention in recent decades for their environmental applications. These oxides are very strong sorbents and oxidants in the environment, capable of removing many contaminants, including organic compounds, heavy metals, and antibiotics. In previous studies, BioMnO_x_ showed better adsorption and degradation of organic and inorganic pollutants, as compared to chemically-synthesized manganese oxides (Cai et al., 2020).

Manganese oxidation is a complex process in Mn-oxidizing bacteria, involving multiple oxidation reactions. Oxidation of soluble Mn(II) ions to Mn(III/IV) oxides has been primarily attributed to direct enzymatic oxidation by microorganisms, such as *Roseobacter* sp. AzwK-3b. This strain can rapidly oxidize Mn(II) to Mn(III) within both cell cultures and cell-free filtrates via enzymatic production of superoxide (Learman et al., 2011a). The exospore of *Bacillus* sp. SG-1 first oxidizes Mn(II) to Mn(III) and then to Mn(IV). MnxG, the most well-studied Mn(II)-oxidizing protein, plays a crucial role in this process. When heterogeneously expressed in *E. coli*, this protein showed Mn(II)-oxidation (Zhou and Fu, 2020) through a coordinated two stage mechanism [Mn(II) → (III) → (IV)] (Soldatova et al., 2017).

The formation of BioMnO_x_ by Mn-oxidizing bacteria involves complex and diverse mechanisms, resulting in various structural variations. Several studies have described BioMnO_x_ as nanoscale phyllomanganates with poor crystallinity, resembling δ-MnO_2_ (Wang et al., 2024, 2017; Meng et al., 2009; Zhou et al., 2015). Several studies have identified BioMnO_x_ in crystalline form, containing as MnO_2_, Mn_3_O_4_, Mn_2_O_3_ or MnCO_3_ phases (Hosseinkhani and Emtiazi, 2011; Wang Y. et al., 2022). A structural transformation of BioMnO_x_ during the formation process have also been documented, for instance, a shift from an initially hexagonal symmetry structure to a final pseudo-orthorhombic structure with improved crystallinity (Learman et al., 2011b). These findings underscore that different Mn-oxidizing bacteria can produce BioMnO_x_ with distinct structural characteristics. As such, detailed investigations are needed to elucidate the factors that influence the structural diversity of BioMnO_x_ among different bacterial strains.

This study, a novel Mn-oxidizing bacterium with strong Mn-oxidation ability was isolated from contaminated soil near an electroplating factory. Unlike Mn-oxidizing bacteria derived from marine environments, which often require specific growth conditions that limit their effectiveness in treating manganese pollution from industrial sources, this soil-derived strain is better suited for such applications. As a result, it is necessary to screen and identify new microbial candidates capable of addressing manganese contamination associated with electroplating activities. Moreover, beyond manganese removal, Mn-oxidizing bacteria can also aid in the remediation of other heavy metal pollutants by forming BioMnO_x_ (Wang M. et al., 2022). These biogenic oxides possess strong adsorption properties, particularly for cadmium metal (Meng et al., 2009; Wang M. et al., 2022; Wang et al., 2024), making them highly effective in co-remediating multiple types of heavy metal contamination.

This study presents a detailed investigation of a newly isolated manganese-oxidizing bacterium. Based on morphological observation, Gram staining, physiological and biochemical characterization, and a 16S rRNA phylogenetic tree, strain M125 was identified as *Lysinibacillus xylanilyticus*. The growth behavior and manganese-oxidizing activity of strain M125 were systematically characterized. and the optimal conditions for biological Mn(II) oxidation were determined. Furthermore, the structures of the BioMnO_x_ produced by this strain were analyzed using a suite of techniques, including transmission electron microscopy (TEM), X-ray diffraction (XRD), X-ray photoelectron spectroscopy (XPS), Fourier transform infrared spectroscopy (FTIR), and energy-dispersive X-ray spectroscopy (EDS). The cadmium adsorption capacity of the bacterial cells (embedded in gel beads) and the manganese oxides were also evaluated. Overall, this work introduces a promising Mn-oxidizing bacterial strain for the bioremediation of manganese-contaminated environments. The study also demonstrates the feasibility of using embedded strains to produce BioMnO_x_ for cadmium removal applications.

## 2 Materials and methods

### 2.1 Culture media, main reagents, and soil samples

The strain was cultured using LB (Luria-Bertani) liquid medium, LB solid medium, and selective medium (prepared by supplementing LB with 10 mmol/L MnCl_2_**·**4H_2_O). Leucoberbelin Blue I (LBB) reagent was purchased from Sigma (Burlington, USA), while MnCl_2_ and KMnO_4_ were sourced from Guoyao Group Co., Ltd (Shanghai, China). Physiological and biochemical test tubes for bacterial identification were purchased from Qingdao Haibo Biotechnology Co., Ltd. Soil samples used for bacterial isolation were collected near an electroplating factory located in Chonglin Industrial Park, Qingdao, Shandong Province, China.

### 2.2 Isolation and screening of Mn-oxidizing bacteria

#### 2.2.1 Isolation and cultivation

Mn-oxidizing bacteria were isolated from the collected soil samples using an enrichment culture approach with a selective liquid LB medium supplemented with manganese chloride (MnCl_2_). The enriched bacterial cultures were serially diluted across six gradients (10^−1^ to 10^−6^), and then plated onto LB agar plates containing MnCl_2_, separately. Individual bacterial colonies were picked and sub-cultured for isolation, purification, and microscopic analysis. Thus, the purified strains were obtained. Finally, the LBB assay was employed to screen and identify strains with strong Mn(II)-oxidation ability (Li et al., 2020; Zhang et al., 2019).

#### 2.2.2 Identification of isolated strains

The selected strains were identified, based on colony morphology, Gram staining, bacterial morphology, physio-biochemical analysis, 16S rRNA sequencing and phylogenetic tree. The 16S rRNA was sequenced at Qingdao RuiBiotech Co., Ltd. The phylogenetic tree was constructed using MEGA-X software (Kumar et al., 2018). The strains were preserved in glycerol at −80 °C.

### 2.3 Growth characteristics of Mn-oxidizing bacteria

#### 2.3.1 Growth curve

Based on the screening results, *Lysinibacillus xylanilyticus* M125 was selected as the Mn-oxidizing bacteria for subsequent experiments. The strain was inoculated into the LB liquid medium for 60 h. After obtaining OD_600nm_ =1, 1% of the culture was inoculated into a fresh LB liquid medium. The automatic growth curve analyzer (Bioscreen C) was used to measure the growth of the strain. The experiment was performed on five parallel groups.

#### 2.3.2 Investigation of the growth characteristics for Mn-oxidizing bacteria M125

The effects of pH, temperature, and Mn^2+^ concentration on the growth of the Mn-oxidizing strain were evaluated by culturing the bacteria under varying conditions and measuring the optical density at 600 nm (OD_600nm_) every 24 h. To assess the effects of pH, the strain was grown at six pH levels (5, 6, 7, 8, 9, and 10), each with three replicates, under constant conditions of 35°C and shaking at 180 rpm using the automatic growth curve analyzer (Bioscreen C). For temperature optimization, shake-flask cultures were incubated at 10°C, 15°C, 20°C, 25°C, 30°C, 35°C, 40°C, and 45°C, maintaining constant pH (7) and shaking speed (180 rpm), with three replicates per temperature. To investigate the effect of Mn^2+^ concentration, the strain was cultured under fixed conditions of pH 7, 35°C, and 180 rpm shaking, with Mn^2+^ concentrations ranging from 10 mM to 80 mM (10, 20, 30, 40, 50, 60, 70, and 80), and three replicates for each concentration. The procedures for strain activation and inoculation are detailed in Section 2.3.1.

### 2.4 Evaluation of the biological oxidation of Mn(II) by Mn-oxidizing bacteria

#### 2.4.1 Analysis of the Mn(II)-oxidizing activity of strain M125

The concentration of BioMnO_x_ produced by the Mn-oxidizing strain was quantified colorimetrically using the LBB assay, as described by Zhu et al. (2016). At regular intervals, 0.1 mL of the culture was mixed with 1 mL of 0.04% LBB in 45 mM acetic acid and reacted in the dark for 5 min. Before absorbance measurement, bacterial cells were removed from the samples via centrifugation. The absorbance of the resulting mixture was then measured at 620 nm using a UV-Vis spectrophotometer. Oxidized LBB produces a blue color, and the degree of this coloration correlates with the amount of Mn oxides present, assuming all oxidized manganese is in the form of MnO_2_ and reduced by LBB. Standard curves prepared with KMnO_4_ showed that the absorbance was linear up to A_620nm_ = 1.50.

#### 2.4.2 Effects of pH, temperature, and Mn^2+^ concentration on Mn^2+^ oxidation by strain M125

The influence of varying pH, temperature, and Mn^2+^ concentration on the biological oxidation of Mn^2+^ by the Mn-oxidizing strain was systematically investigated. The bacterial strain was activated as outlined in Section 2.3.1. Following adjustment of the culture to an OD_600nm_ of 1.0, 1% (v/v) of the inoculum was introduced into an LB liquid medium. The Mn(II) oxidation experiments were conducted by altering one parameter at a time: pH levels (5, 6, 7, 8, 9, and 10), temperatures (15°C, 20°C, 25°C, 30°C, 35°C, and 40°C), and Mn^2+^ concentrations (6 mM, 12 mM, 18 mM, and 24 mM). Three replicates were set for each experimental group, as well as for the control group without bacterial inoculation. Unless otherwise specified, the default culture conditions were pH 7.0, temperature 35°C, Mn^2+^ concentration 6 mM, and shaking speed 180 rpm. Samples were collected every 24 h. The Mn(II)-oxidizing activity of the strain was measured as described in Section 2.4.1. Based on the absorbance value at 620 nm, the concentration of oxidized manganese in the culture solution was measured. Simultaneously, bacterial growth was monitored via OD_600nm_ measurements at 24 h intervals.

### 2.5 Collection of BioMnO_*x*_ from the culture

Strain M125 was cultured in LB liquid medium supplemented with 6 mM Mn^2+^ under pH 7.0, 35°C conditions and shaking at 180 rpm for 14 or 23 days. Following incubation, the bacterial suspension was subjected to ultrasonic disruption for 1 h to lyse the cells. The resulting lysate was centrifuged at 4°C and 2,000–3,000 rpm for 25 min. After discarding the supernatant, the pellet was re-suspended in ultrapure water, thoroughly mixed, and centrifuged at 4°C and 4,000 rpm for 15 min. This washing step was repeated three times. The final pellet obtained was collected as BioMnO_x_.

### 2.6 Characterization of BioMnO_*x*_

#### 2.6.1 TEM analysis

Bacterial morphology analysis: cells of *Lysinibacillus xylanilyticus* M125 in the log-growth phase were collected by centrifugation. After washing, cell pellets were re-suspended in 0.1 M phosphate-buffered saline (pH 7.4). A small cell suspension was applied onto a copper grid coated with amorphous carbon film and negatively stained using phosphotungstic acid (pH 6.5) for 1 min. The morphology of stained bacterial cells was investigated with a Tecnai G2 F20 TEM.

Characterization of BioMnO_x_: BioMnO_x_ samples were diluted to an appropriate concentration and deposited onto carbon coated grids for TEM analysis. In parallel, a mixture of bacterial cells and BioMnO_x_ was prepared by centrifuging a 23-day bacterial culture grown in the presence of Mn^2+^. And this mixture was also subjected to TEM observation. Imaging was performed using a Tecnai G2 F20 TEM, while elemental analysis was carried out using a Talos F20X TEM (Thermo Fisher). Both instruments operated at an accelerating voltage of 200 kV. Elemental composition was analyzed using the EDS in scanning TEM mode with four detectors.

#### 2.6.2 Macroscopic analysis of BioMnO_*x*_

The BioMnO_x_ were collected (as described in Section 2.5), washed, and dried to obtain a powder form. The powdered BioMnO_X_ samples were then characterized by XRD, XPS, and FTIR. XRD was performed using a Rigaku Smart Lab 9 kW instrument with Cu Kα (1.542 Å radiation). XPS analysis was carried out using a Thermo Fisher ESCALAB 250XI XPS, and the resulting data were analyzed by Avantage 5.9. FTIR spectra were acquired through a Bruker VERTEX 70 v spectrometer with 4 cm^−1^ resolution, at wavenumber ranging from 1,000 to 4,000 cm^−1^.

### 2.7 Cd-adsorption capacity of BioMnO_*x*_

#### 2.7.1 Embedding of bacterial strain in gel beads

Preparation of Bacteria-Embedded Gel Beads: Strain M125 was cultured in 200 mL of liquid LB medium at pH 7, 35°C and 180 rpm for 2 days. The culture, harvested during the logarithmic growth phase, was used as the seed solution for embedding. The OD_600nm_ of the culture was adjusted to 1.0 using fresh LB medium. Subsequently, 5 mL of the adjusted bacterial suspension was mixed with 25 mL of 2% (w/v) sodium alginate solution. This mixture was then dispensed dropwise into a culture dish containing ~30 mL of 5% (w/v) CaCl_2_ solution from a height of ~30 cm using a syringe. This culture dish was stored at 4°C for 24 h to allow complete gelation and immobilization of the bacterial cells. The resulting bacteria-entrapped gel beads were washed thoroughly with ultra-pure water several times before further use.

#### 2.7.2 Measurement of Cd adsorption

Cd-adsorption capacity was measured for three types of samples (M125, embedded M125, and BioMnO_x_-M125). For the Cd-adsorption experiment using M125: M125 strain was activated as described earlier (Section 2.4.1). After adjusting the OD_600nm_ to 1.0, 2.5% of the M125 culture was inoculated into 200 mL of LB liquid medium (pH 7.0) supplemented with 1 mM Cd^2+^ and 6 mM Mn^2+^. The culture was incubated at 35°C with shaking at 180 rpm for 10 days. For the Cd-adsorption experiment using embedded M125: Gel beads containing immobilized M125 cells were added to 200 mL of LB liquid medium (pH 7.0) containing the same concentrations of Cd^2+^ (1 mM) and Mn^2+^ (6 mM), and incubated under identical conditions (35°C and 180 rpm) for 10 days. After 10 days of cultivation, both cultures were centrifuged, and the supernatants were collected for analysis. All experimental groups were performed in triplicate, while the control group (without bacterial inoculation) was included for comparison.

For the BioMnO_x_-M125 adsorption test, 1.5 g of BioMnO_x_ (harvested from a 14-days M125 culture as described in Section 2.5) was added to 200 mL of fresh LB medium containing 1 mM Cd^2+^. The mixture was incubated at 35 °C and 180 rpm for 2 days, after which the supernatant was collected by centrifugation.

The above three supernatant samples were filtered through 0.22 μm membranes, acidified with 1% HNO_3_, and stored at 4°C for cadmium analysis using an inductively coupled plasma mass spectrometer (ICP-MS, PerkinElmer 1000G). The residual Cd^2+^ concentrations in the supernatants were used to evaluate the cadmium adsorption capacities of M125 strain, M125-embedded gel beads, and BioMnO_x_-M125.

## 3 Results

### 3.1 Screening and identification of bacterial strains

A total of 47 bacterial strains were isolated from the collected soil samples. Among them, 11 strains showing high manganese tolerance and brownish colony coloration were selected through preliminary screening due to the formation of biogenic manganese oxides, details about these strains can be seen in Supplementary Table S1. Strain M125 was ultimately chosen for further investigation due to its improved manganese oxidizing capacity.

When strain M125 was cultured in the selective medium (initial pH 7.0), the medium turned light brown at first, and then gradually turned dark as the cultivation time prolonged. Supplementary Figure S1a shows the 13-d bacterial culture obtained after inoculating M125 into liquid LB medium containing 6 mM Mn^2+^. The liquid culture showed a typical brown color of BioMnO_x_. It is well known that LBB oxidizes and turns blue in the presence of Mn (Mn^3+^, Mn^4+^, or Mn^7+^) under weak acid conditions, resulting in a bright blue solution. The test tube indicated by the arrow in Supplementary Figure S1a shows the typical bright blue solution obtained after the LBB assay. This confirmed the production of BioMnO_x_ by strain M125, which oxidized the LBB, generating blue color. The control group with no bacterium inoculation (Supplementary Figure S1b) showed very low concentration of Mn-oxides in the solution.

**Figure 1:**
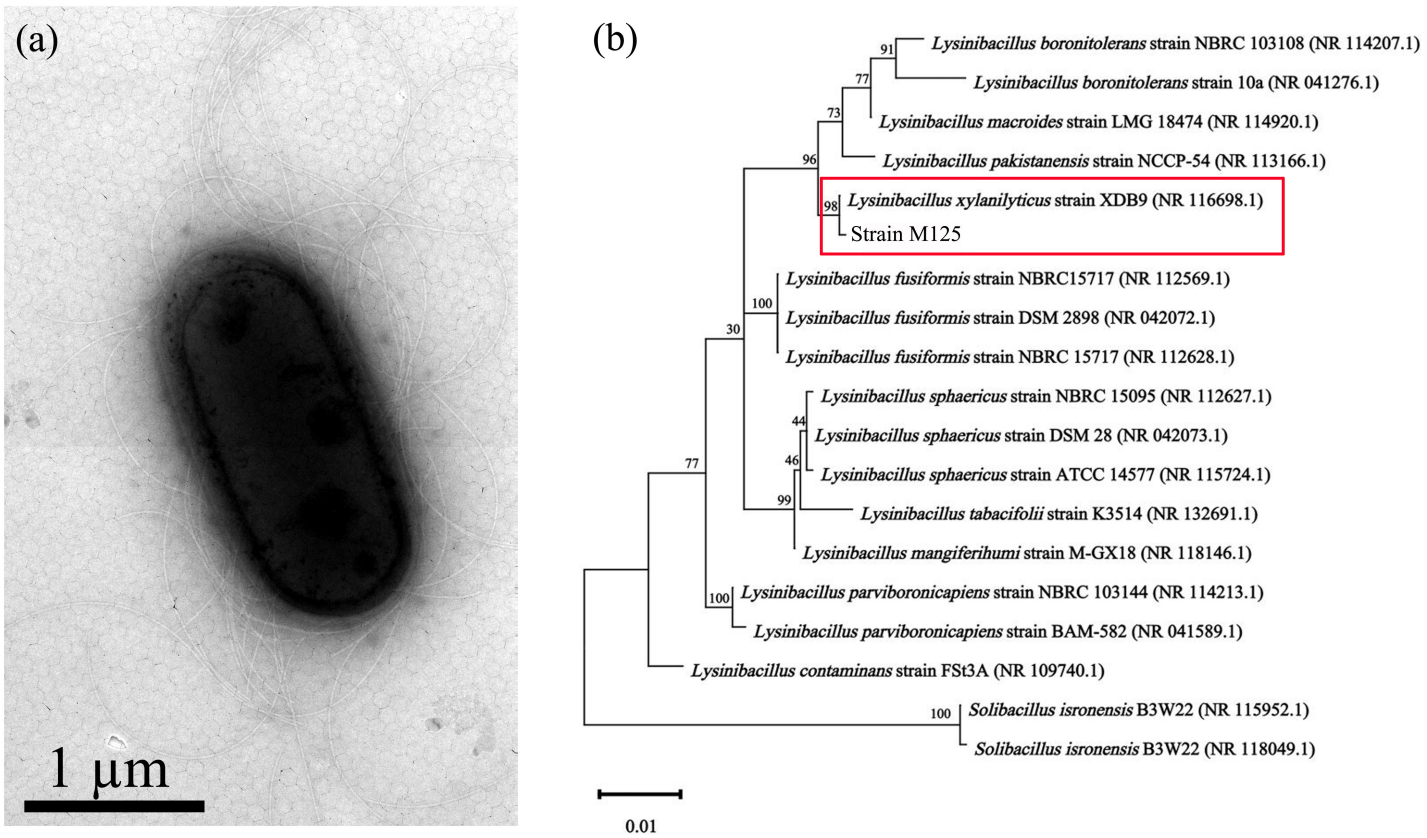
Mn-oxidizing *Lysinibacillus xylanilyticus* M125 strain isolated from the soil near electroplating factory: **(a)** TEM morphology and **(b)** Phylogenetic tree based on 16S rRNA sequencing.

The morphology of M125 bacterial strain was examined by TEM. It is a rod-shaped bacterium (measuring 2.0–2.2 μm × 0.9–1.1 μm), with peritrichous flagella (Figure 1a). Phylogenetic analysis based on 16S rRNA gene sequencing indicated that M125 shared high similarity (> 98% identity) with the sequence data of *Lysinibacillus xylanilyticus* strains procured from the openly accessible database (Figure 1b). Accordingly, the strain M125 was identified as *Lysinibacillus xylanilyticus* M125. The physiological and biochemical characteristics of M125 were further assessed, and the results are summarized in Table 1.

**Table 1:** Results of physio-biochemical analysis and gram staining of strain M125 (+ is positive result; – is negative result).

**Physio-biochemical reactions**	**Results**	**Physio-biochemical reactions**	**Results**
Urea enzymes	+	MR-VP	–
H_2_S	–	Maltobiose	–
Gelatin liquefaction	–	Glucose	–
Arabinose	+	Malonate	–
Mannitol	–	3% H_2_O_2_	+
Fructopyranose	–	Dynpower culture	–
Saccharose	–	Nitrate reduction	
Lactobiose	–	Litmus milk	–
Simoncitrate	–	Galactose	–
Wood sugar	+	Gram stain	+

### 3.2 Growth characteristics of strain M125

#### 3.2.1 Growth curve

**Figure 2:**
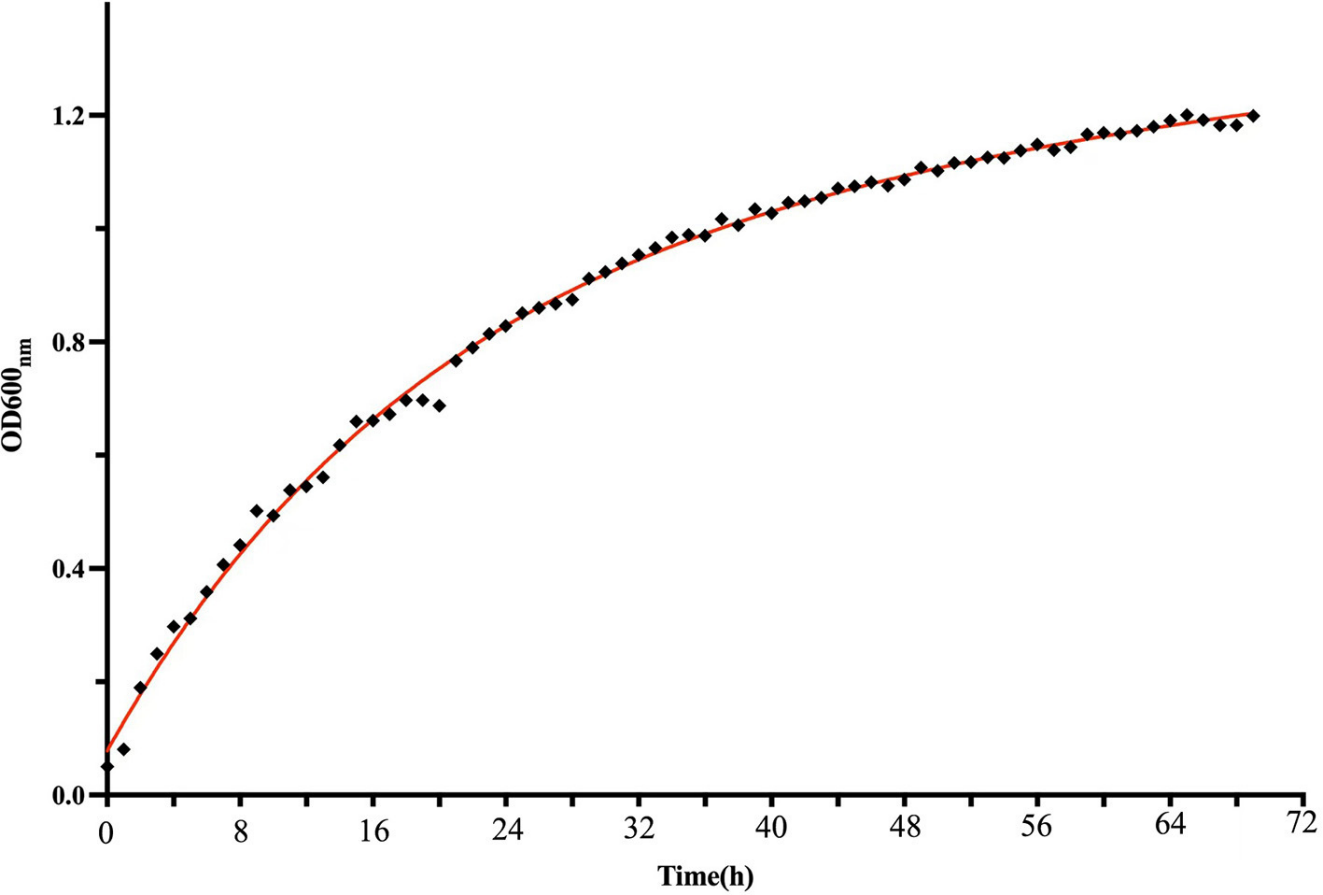
The growth curve of *Lysinibacillus xylanilyticus* M125 strain.

The growth dynamics of strain M125 were monitored using the automatic growth curve analyzer (Bioscreen C), and the results are presented in Figure 2. Strain M125 showed a rapid exponential growth phase during the first 60 h of cultivation, after which it transitioned into a stationary phase. After 60 h of cultivation, the OD_600nm_ value of strain M125 gradually stabilized within the range of 1.0 to 1.2. These findings showed the fast growth rate of strain M125, underscoring its potential for practical applications.

#### 3.2.2 Effects of temperature, Mn^2+^ concentration, and pH on the growth of strain M125

**Figure 3:**
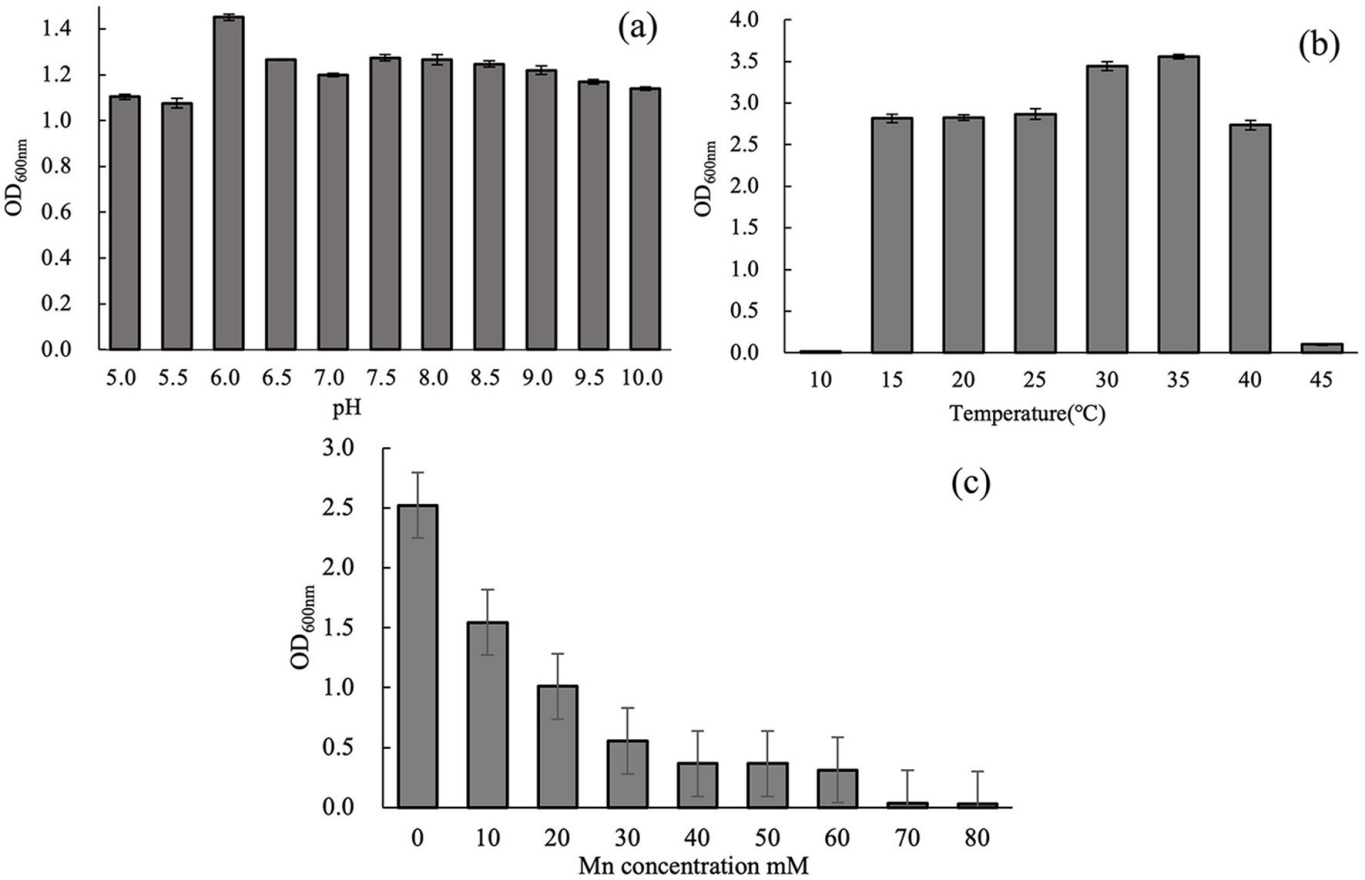
Effects of **(a)** pH (48 h culture), **(b)** temperature (72 h culture), and **(c)** Mn^2+^ concentration (48 h culture) on the growth of strain M125.

Figure 3a illustrates the effect of pH on the growth of strain M125 after 48 h of cultivation. The strain M125 can grow well across a broad pH range (5.0–10.0), indicating its strong adaptability and tolerance to varying pH conditions. This suggests that M125 can thrive in diverse environmental settings. Figure 3b presents the influence of temperature on M125 growth over 72 h. Strain M125 hardly grew at temperatures lower than 10°C, or higher than 45°C. It showed good growth within the temperature range of 15°C to 40°C. Optimal growth was observed in the 30°C−35°C range, identifying this interval as the preferred temperature for bacterial cultivation. These results indicate that the strain M125 has considerable temperature tolerance. Figure 3c shows the effect of Mn^2+^ concentration on bacterial growth after 48 h. Increasing Mn^2+^ levels gradually inhibited growth, with complete suppression observed at concentrations above 60 mM. As it can grow within the Mn^2+^ concentration range of 0 to 60 mM, indicating that the Mn^2+^ tolerance of this strain was up to 60 mM. These findings demonstrate that M125 possesses a remarkably high Mn^2+^ resistance, suggesting the potential applications of strain M125 in serious manganese-contaminated environments, such as bioremediation processes.

### 3.3 Conditions of biological oxidation of Mn^2+^ by strain M125

**Figure 4:**
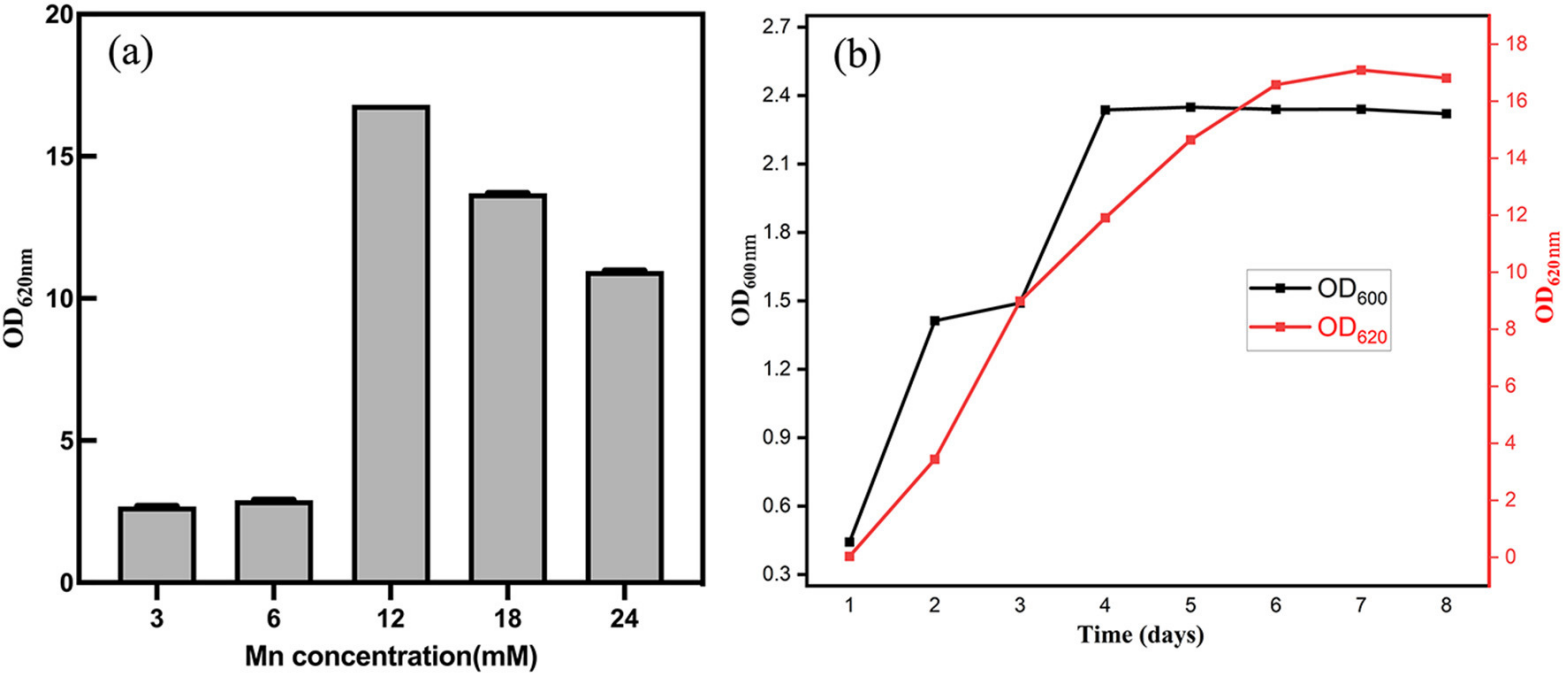
**(a)** After cultivation for 8 d, OD_620 nm_ values indicating the levels of Mn oxidation at different concentration of Mn^2+^. **(b)** Cell growth (based on OD_600 nm_) and manganese oxidation (based on OD_620 nm_) at pH 7 with Mn^2+^ concentration of 12 mM.

Different Mn^2+^ concentrations significantly influenced the manganese-oxidizing capacity of strain M125. As shown in Figure 4a, Mn oxidation levels reflected by OD_620__nm_ values increased with rising Mn^2+^ concentrations up to a certain threshold. Mn-oxidizing activity improved with increasing Mn^2+^ levels but declined when concentrations exceeded 12 mM. Figure 4b illustrates the growth (OD_600nm_) and manganese oxide production (OD_620__nm_) of strain M125 in an LB medium containing 12 mM Mn^2+^ at pH 7. The result shows that the value of OD_620nm_ would rapidly increase as time increase, illustrating that the manganese(II) ions are gradually oxidized into high valence manganese states by the bacterium.

The different pH also influences the Mn-oxidation ability. Supplementary Figure S2 shows the levels of Mn oxidation at different pH, indicated by the OD_620nm_. The experimental results show that the Mn-oxidation ability of strain M125 enhanced under higher pH conditions. This is because a higher pH (weak alkaline condition) is conducive to abiotic Mn(II) oxidation. However, the high pH is detrimental to the activity of bacterial enzymes that mediate biotic Mn(II) oxidation (Comert and Tepe, 2020). These results suggest that although higher pH favors chemical oxidation, it may inhibit bacterial enzyme-mediated processes.

To ensure complete oxidation of Mn(II) ions by strain M125, 6 mM was chosen as the Mn^2+^ concentration. Also, to provide the maximum activity of bacterial enzymes for biological manganese oxidation, an initial pH of 7.0 was chosen. For subsequent experiments, the Mn-oxidation ability of strain was determined at 35°C temperature, shaking speed of 180 rpm, pH 7.0, and Mn^2+^ concentration of 6 mM.

### 3.4 Characterization of BioMnO_*x*_

**Figure 5:**
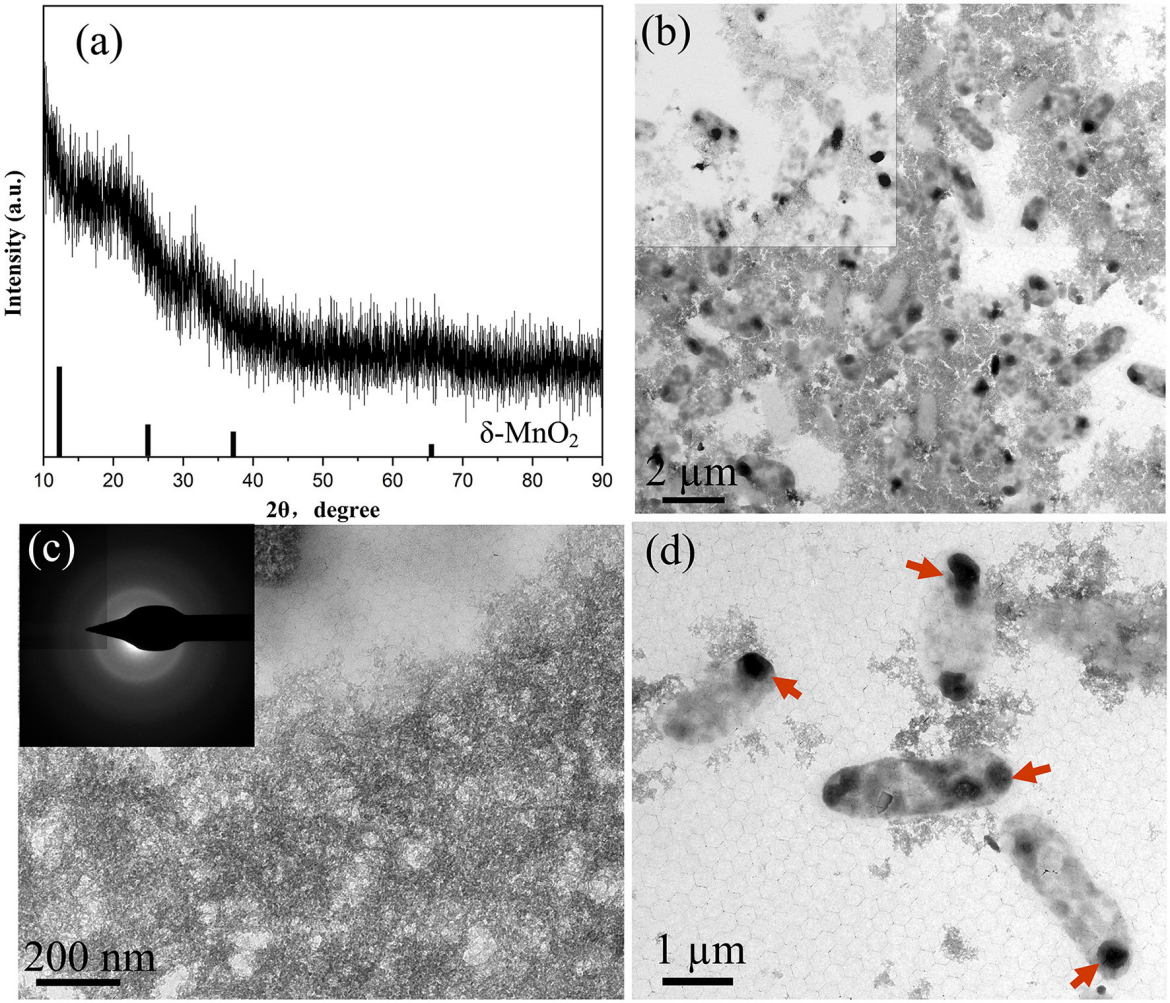
Characterization of BioMnO_x_ obtained after 23-d culturing of strain with Mn^2+^: **(a)** XRD pattern; **(b)** TEM low-magnification image; **(c)** Enlarged TEM image of nanosheet BioMnO_x_ and corresponding SAED pattern; **(d)** Enlarged TEM image of globular BioMnO_x_, with BioMnO_x_ marked by red arrows.

**Figure 6:**
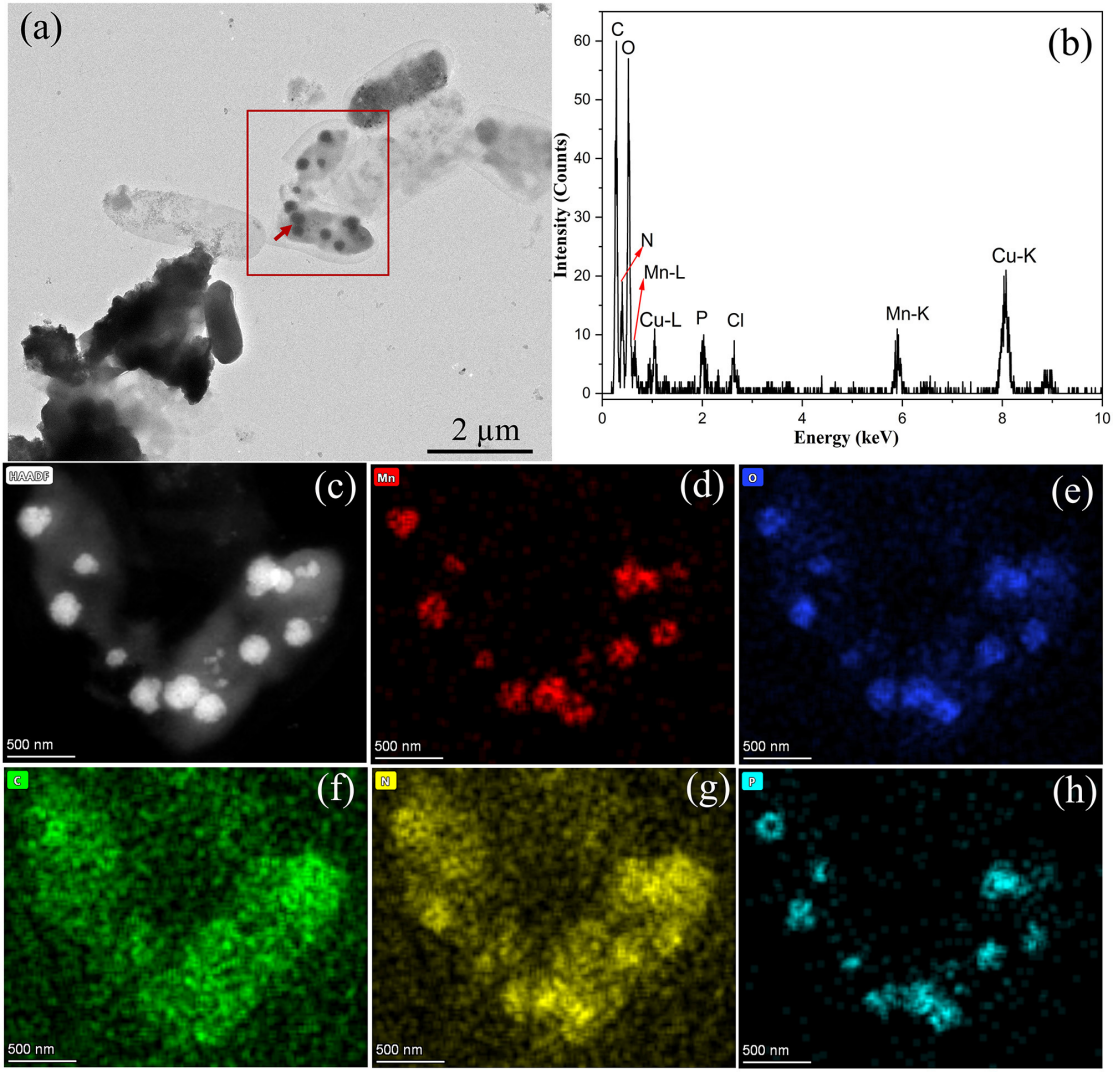
Characterization of BioMnO_x_ produced by strain M125: **(a)** TEM image of globular BioMnO_x_ marked with red arrow; **(b)** EDS spectrum of globular BioMnO_x_; **(c)** HAADF image; **(d–h)** EDS maps of Mn, O, C, N, and P, respectively.

In this study, the structural characteristics of BioMnO_x_ were comprehensively analyzed, with particular attention given to its structural evolution over the cultivation period. Figure 5 presents the XRD patterns and TEM images of BioMnO_x_ collected obtained after 23 days of bacterial cultivation in the presence of Mn^2+^. As shown in Figure 5a, the XRD pattern reveals that the BioMnO_x_ primarily revealed an amorphous structure, aligning with previous findings that BioMnO_x_ produced by microorganisms tends to be poorly crystalline (Villalobos et al., 2003). The low-magnification TEM image in Figure 5b highlights two predominant morphologies: nanosheets and globular forms. High-magnification images in Figures 5c, d provide a closer view of the ultrathin nanosheets and the globular aggregates enclosed within bacterial sheaths. The selected area electron diffraction (SAED) pattern inserted in Figure 5c confirms the amorphous nature of the nanosheets, resembling the nanostructured, todorokite-like porous structure of BioMnO_x_ described in previous studies (Meng et al., 2009). In Figure 5d, the globular BioMnO_x_ structures are indicated by red arrows. Further elemental composition analysis of the globular form is presented in Figure 6.

The globular BioMnO_x_ particles, ~200–300 nm in diameter, were encapsulated by bacterial sheaths. Figure 6b shows the EDS spectrum of globular BioMnO_x_ marked with red arrow in Figure 6a. The EDS spectrum indicates the presence of a high content of Mn element and a substantial amount of oxygen in globular BioMnO_x_, confirming that the particles were manganese oxides. Further analysis of these globular particles was conducted using scanning TEM mode. Figures 6c–h present the results for the red-rectangle region highlighted in Figure 6a, including a high-angle annular dark field (HAADF) image and elemental distribution maps for Mn, O, C, N, and P. The EDS maps show that the globular nanoparticles consisted primarily of Mn and O elements, consistent with manganese oxides. Moreover, N and P elemental contents were also relatively high. These findings suggest that the BioMnO_x_ synthesized by strain M125 is not a purely inorganic manganese oxide but an organic-inorganic composite incorporating manganese within a biological matrix.

**Figure 7:**
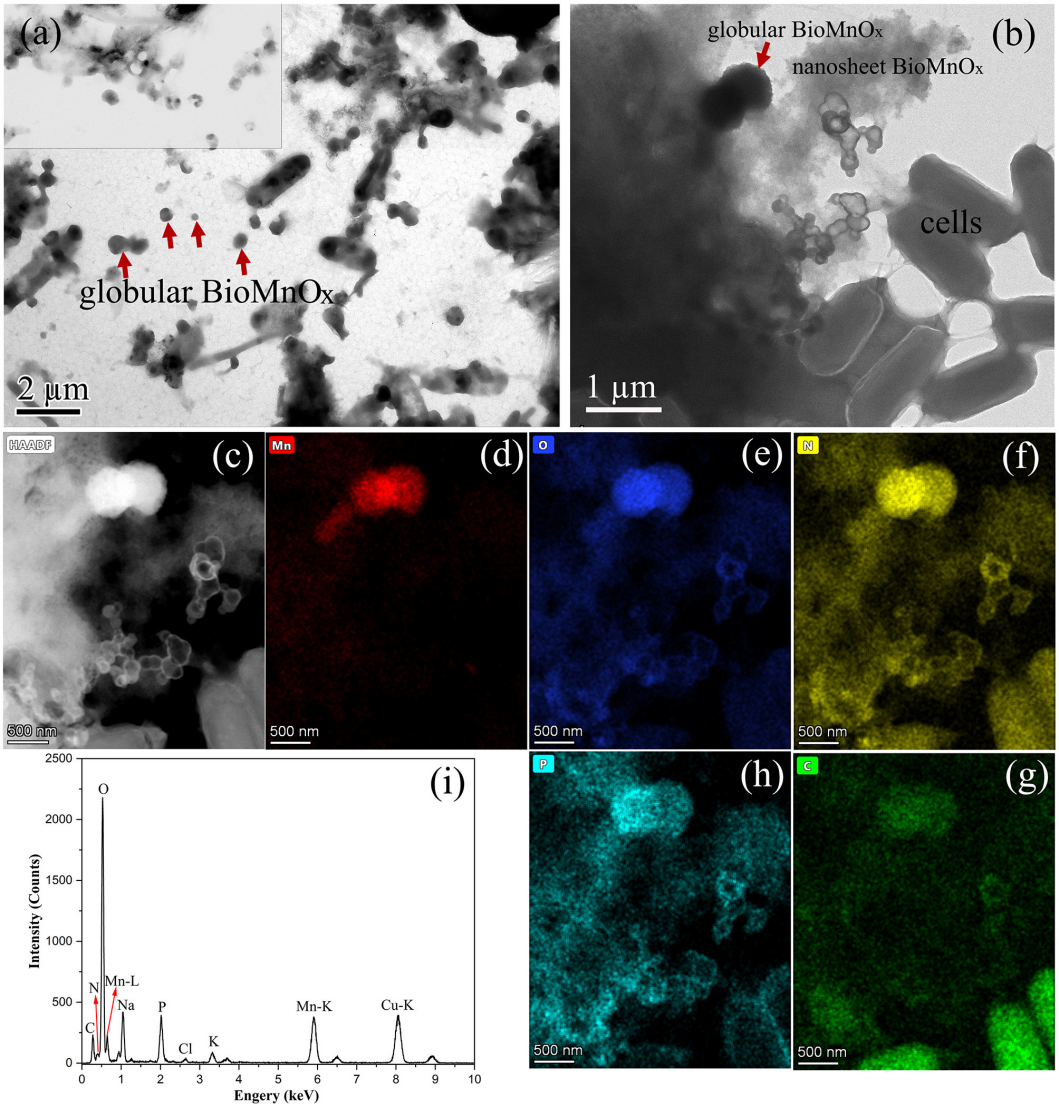
Characterization of strain 125 after 23-d culture: **(a)** Low-magnification TEM image with large number of extracellular globular BioMnO_x_; **(b)** TEM image with an extracellular globular BioMnO_x_ embedded in nanosheet BioMnO_x_, marked by red arrow; **(c)** HAADF image; **(d–h)** corresponding EDS maps of Mn, O, N, C, and P, respectively; and **(i)** EDS spectrum of extracellular globular BioMnO_x_ marked by red arrow in Figure b.

In addition to the isolated BioMnO_x_, strain M125 cultured with Mn^2+^ for 23 days was also investigated by TEM, as shown in Figure 7. A substantial amount of extracellular globular BioMnO_x_ was observed within the bacterial culture. Figures 7a, b present the low- and high-magnification TEM images of strain M125, with red arrows pointing to the extracellular globular BioMnO_x_. Unlike the sheath-wrapped globular BioMnO_x_ observed in Figure 6, these globular particles appeared free-standing and lacked encapsulation. Further analysis of these extracellular globular BioMnO_x_ was conducted using EDS, as shown in Figures 7c–i. Figures 7c–h shows the HAADF image and elemental distribution maps for Mn, O, C, N, and P for the globular particles identified in Figure 7b. These figures confirm the presence of Mn and O elements in the extracellular globular BioMnO_x_. Figure 7i shows the EDS spectrum of globular BioMnO_x_ marked with a red arrow in Figure 7b, which further confirmed the presence of a large amount of Mn and O elements in BioMnO_x_. All these results implied that these nanoparticles were indeed manganese oxides. The observed characteristics of these globular BioMnO_x_ particles are consistent with previous findings. For instance, according to Su et al., BioMnO_x_ has a distinct polyhedral structure with globular nanoparticles of 150–350 nm diameters, which are the catalytic products of CueO enzymes (Su et al., 2014). Huang et al. reported the formation of globular BioMnO_x_ via cocultures under anaerobic microbial oxidation of manganese (Huang et al., 2023).

**Figure 8:**
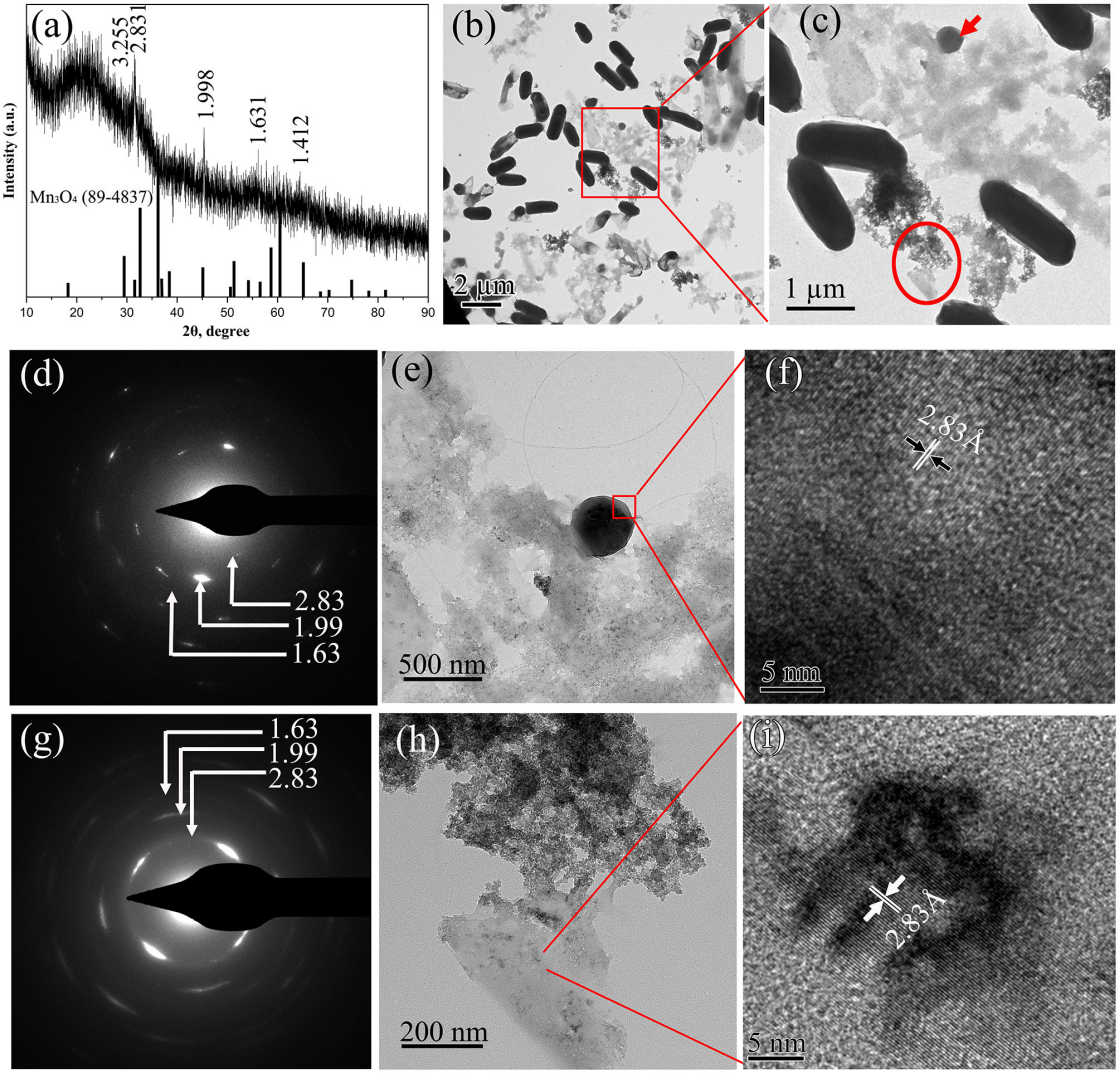
Characterization of BioMnO_x_ obtained after 90-d cultivation of strain with Mn^2+^: **(a)** XRD pattern; **(b)** Low-magnification TEM image; and **(c)** the enlarged TEM image of the region marked by red rectangle, displaying globular (red arrow) and nanosheet (red circle) forms of BioMnO_x._; **(d)** SAED pattern; **(e)** Enlarged TEM image; **(f)** HRTEM image of globular BioMnO_x_; **(g)** SAED pattern; **(h)** Enlarged TEM image; and **(i)** HRTEM image of nanosheet BioMnO_x_.

The structural evolution of BioMnO_x_ synthesized by strain M125 over time was also investigated. Figure 8 illustrates the characteristics of BioMnO_x_ produced after 90 days of cultivation. Compared to the XRD pattern obtained at 23 days (Figure 5a), the 90-day XRD profile (Figure 8a) revealed a transformation from an amorphous to a more crystalline phase. Several diffraction peaks corresponded to Mn_3_O_4_ (PDF#89-4837), while some broad peaks characteristic of amorphous MnO_2_ remained, indicating the coexistence of both Mn_3_O_4_ and MnO_2_ phases in the mature BioMnO_x_. More pronounced crystalline peaks were observed when the cultivation temperature was higher (about 40°C) (Supplementary Figure S4). TEM analysis (Figures 8b–i) also supported the presence of two distinct morphologies throughout the structural transition. A low-magnification TEM image (Figure 8b) shows the bacterial cells, nanosheet, and globular-shaped BioMnO_x_ particles together. Further structural analysis using SAED and HRTEM confirmed the crystalline nature of these forms. The SAED pattern of the globular BioMnO_x_ (Figure 8d) showed clear Bragg diffraction rings, with d spacing of 2.83 Å, 1.99 Å, and 1.63 Å. These diffraction rings in the SAED patterns were consistent with the diffraction peaks observed in XRD pattern, revealing that the globular BioMnO_x_ consisted of Mn_3_O_4_. In the corresponding HRTEM image, globular BioMnOx had a uniform lattice fringes with an interplanar spacing of 2.83 Å. The nanosheet BioMnO_x_ displayed a mixture of amorphous and crystalline regions (Figures 8g–i), with the crystalline domains resembling the Mn_3_O_4_ phase. These findings confirm the simultaneous presence of MnO_2_ and Mn_3_O_4_ in BioMnO_x_ and highlight the progressive structural maturation during extended cultivation.

**Figure 9:**
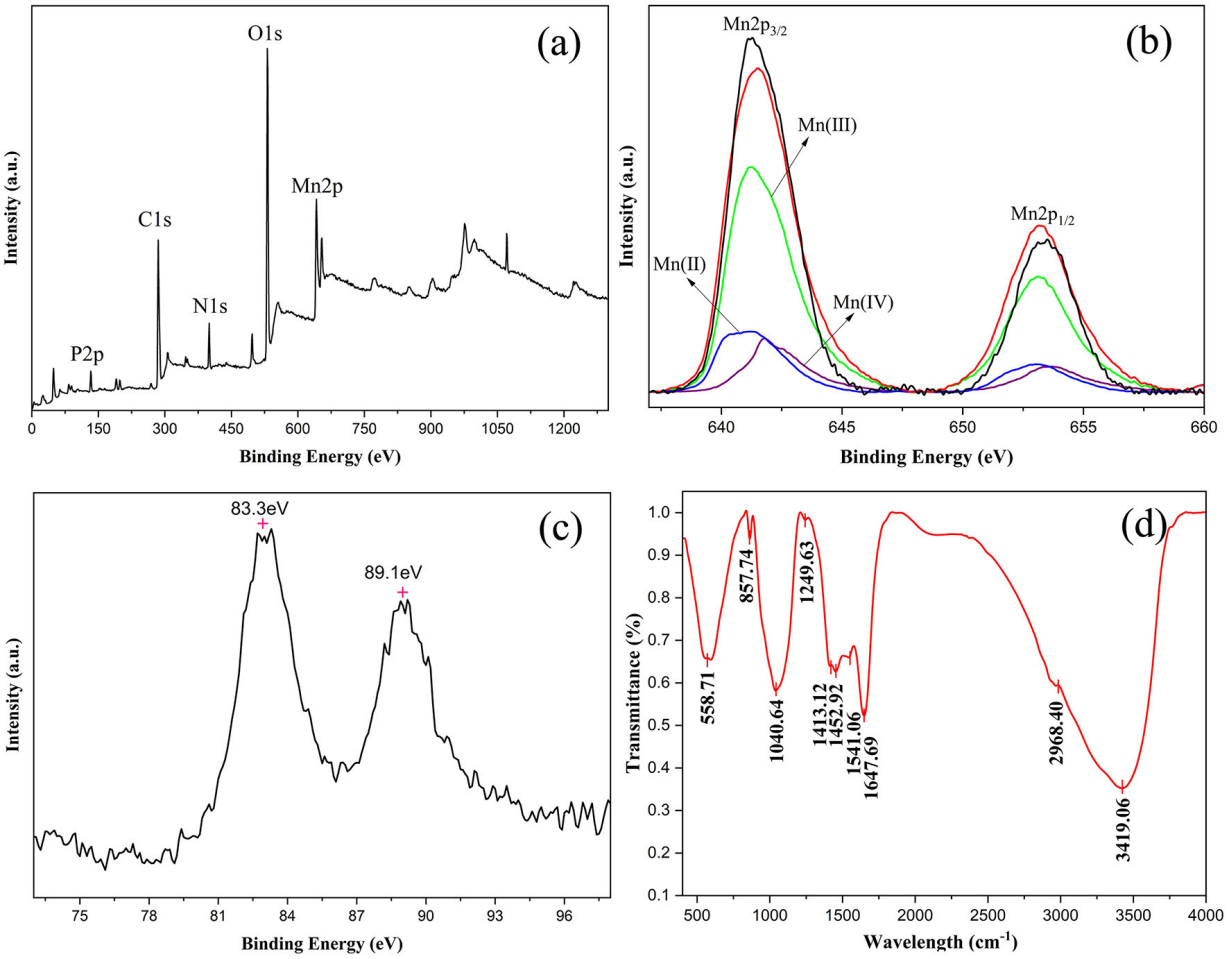
Characterization of BioMnO_x_ collected after cultivation of strain M125 for 23 d: **(a)** XPS spectrum; **(b)** XPS spectrum of Mn 2p; **(c)** XPS spectrum of Mn 3s; and **(d)** FTIR spectrum of BioMnO_x_.

The BioMnO_x_ samples were further analyzed using XPS and Fourier-transform infrared spectroscopy (FTIR) to elucidate their elemental composition and chemical structure. XPS analysis confirmed the presence of C 1s, N 1s, O 1s, P 2p, and Mn 2p (Figure 9a), indicating that carbon, oxygen, nitrogen, phosphorus, and manganese were the principal elements in BioMnO_x_. This finding was consistent with the EDS results (shown in Figures 6, 7). The deconvolution of the Mn 2p spectrum revealed binding energies at 641.2 eV and 653.3 eV, which correspond to Mn^2+^, Mn^3+^, and Mn^4+^ oxidation states (Figure 9b). Furthermore, the Mn 3s peak, which shows spin-orbit splitting, was used to evaluate manganese valence states further. This peak has two split components. Magnitude of peak splitting indicates the oxidation state (Biesinger et al., 2011). The ΔE of the Mn 3s peak of BioMnO_x_ was about 5.8 eV (Figure 9c), indicating that the BioMnO_x_ contained various valence states of Mn, such as MnO_2_, Mn_2_O_3_ etc. FTIR spectroscopy (Figure 9d) of BioMnO_x_ provided further structural insight. The broad and intense absorption band at 3,419 cm^−1^ corresponds to O–H stretching, indicative of extensive hydrogen bonding. The strong band at 2,968 cm^−1^ is associated with –CH_3_ groups. Sharp absorption peaks at 1,647 cm^−1^ and 1,541 cm^−1^ correspond to amide and –NO_2_ functional groups, respectively. Together, these results suggest that the BioMnO_x_ produced by strain M125 is not merely an inorganic manganese oxide but a complex organo-mineral composite incorporating manganese within an organic matrix.

### 3.5 Cd-adsorption capacity of BioMnO_*x*_

**Figure 10:**
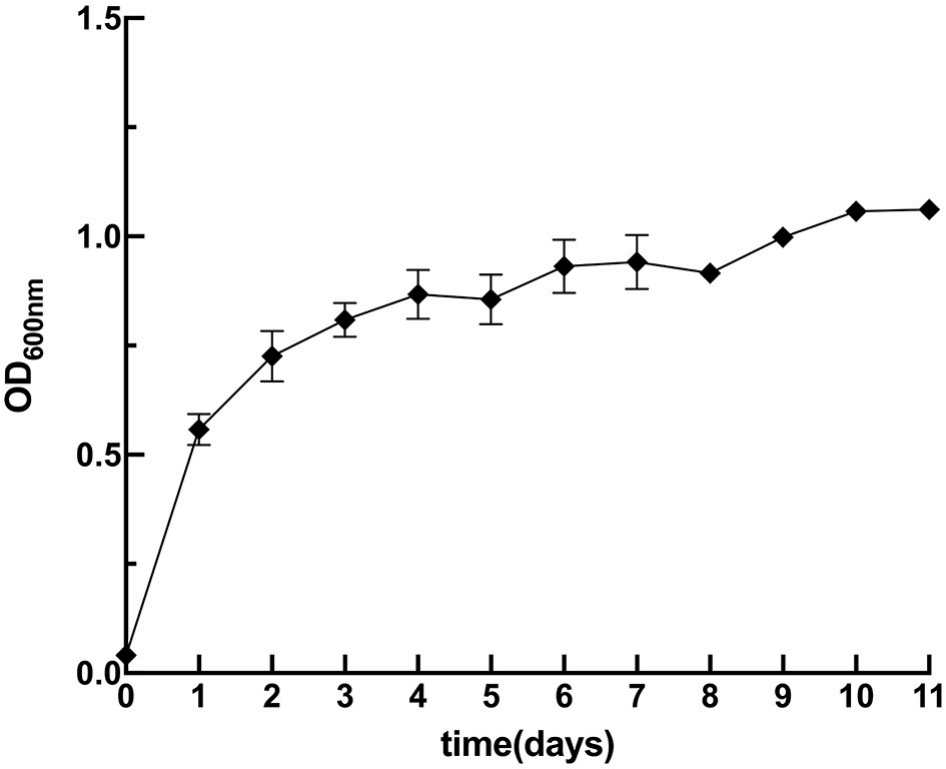
Growth curve of M125 strain in liquid LB containing 1 mM Cd ions.

BioMnO_x_ is known for its strong adsorption capacity and has been explored for the remediation of heavy metal contamination (Wang M. et al., 2022). This study evaluated the Cd-adsorption ability of BioMnO_x_ produced by strain M125. Firstly, the Cd tolerance of strain M125 was assessed. As shown in Figure 10, the strain showed robust growth in liquid LB medium supplemented with 1 mM Cd^2+^, indicating that the strain grew well and had a good adaptability to cadmium stress.

To mimic conditions suitable for practical application, strain M125 was immobilized in gel beads. Supplementary Figures S5a, b display the gel beads with 25% and 35% (vol.) bacterial embedding, respectively. After 24 h of mechanical testing, the beads with 35% embedding showed deformation and cracking. Therefore, 25% embedded of gel beads was selected for the subsequent experiments.

**Figure 11:**
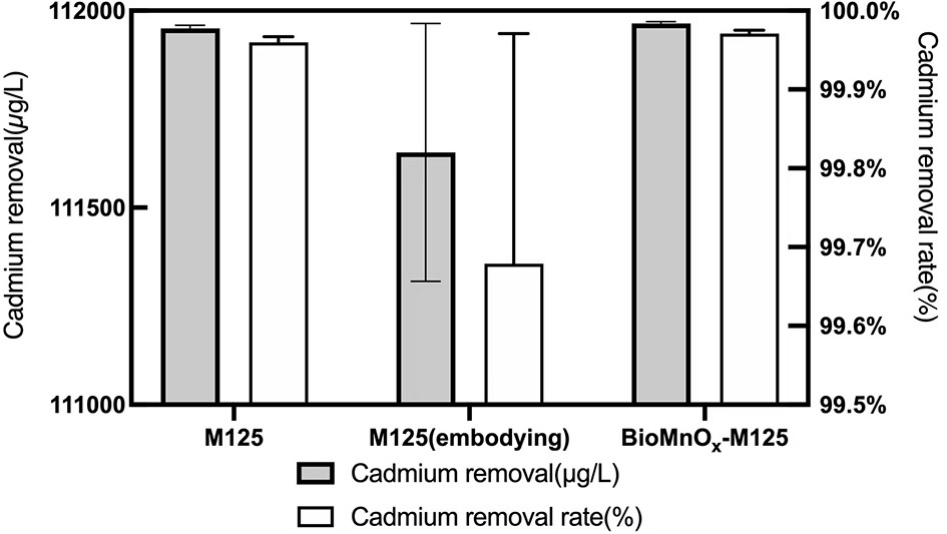
Concentration of residual Cd in the supernatants, and Cd-removal rates of the three samples.

Both strain M125 and embedded M125 were cultured in LB liquid medium containing 6 mM MnCl_2_ and 1 mM CdCl_2_ at 35°C and 180 rpm for 10 days. Following cultivation, both cultures were centrifuged to collect the supernatants. And the residual Cd concentrations in the supernatants were measured, the results are shown in Figure 11. Furthermore, the Cd-adsorption efficiency of purified BioMnO_x_-M125 was also investigated. The BioMnO_x_ was incubated in an aqueous solution containing 1 mM Cd^2+^ for 2 days, and the Cd concentration in the supernatant was subsequently determined. The Cd removal rates of M125, embedded M125, and BioMnO_x_-M125 were 99.96%, 99.52%, and 99.97%, respectively.

These findings demonstrate that strain M125 and its BioMnO_x_ product possess excellent Cd-adsorption capabilities. The embedded M125 form also maintained high adsorption performance, highlighting the promising potential of strain M125 for the bioremediation of environments co-contaminated with manganese and cadmium.

## 4 Discussion

In this study, a novel manganese-oxidizing bacterial strain was obtained from the contaminated soil near an electroplating factory. Based on 16S rRNA sequencing and physiological-biochemical analysis (Figure 1; Table 1), the strain was identified as Lysinibacillus *xylanilyticus*. Assessment of the effects of varying pH, temperature, and Mn^2+^ concentrations on its growth revealed that the strain M125 thrives across a broad pH range of 5.0–10.0, temperature range of 15°C−40 °C, and Mn^2+^ concentrations up to 60 mM (Figure 3).

The factors influencing the Mn-oxidizing capacity of strain M125 were systematically investigated. The strain demonstrated robust growth and manganese-oxidizing activity within a pH range of 5.0–9.0. It could also grow and oxidize Mn^2+^ at concentrations ranging from 3 to 24 mM. And its Mn-oxidation ability was stronger when Mn^2+^ concentration was in the range of 12–18 mM (Figure 4). These results indicate that the strain M125 shows rapid growth and tolerance to a wide range of pH, temperature, and Mn^2+^ concentrations, highlighting its promising potential for practical applications.

BioMnO_x_ produced by strain M125 via Mn oxidation was comprehensively characterized using TEM, XRD, XPS, and FTIR analyses. The XRD pattern of BioMnO_x_ collected after 10–20 days of cultivation indicated a predominantly amorphous structure (Figure 5a). TEM observation revealed a structurally complex BioMnO_x_. There were two forms of BioMnO_x_: nanosheets and globular particles (Figures 5b–d). The nanosheet BioMnO_x_ was primarily amorphous, as revealed by its SAED pattern. Some previous reports have found that the BioMnO_x_ produced by Mn-oxidizing bacteria, such as *L. discophora* SP-6, and *P. putida* MnB1, are porous MnO_2_ with a poorly crystalline structure (short-ranged or amorphous), and the nano-sized BioMnO_x_ are roughly 8–9 nm in size (Meng et al., 2009). The morphology and scale of these nanosheet BioMnO_x_ observed in this study were consistent with these previously described porous MnO_2_ structures.

In addition to the nanosheet structures, TEM analysis revealed many globular BioMnO_x_ particles. These globular forms appeared in two distinct types: some were encapsulated by a bacterial sheath (Figure 6), while others were freely dispersed as extracellular particles (Figure 7). TEM observation of the strain M125 cultured without Mn^2+^ (Supplementary Figure S3) showed a clear sheath structure on the cell surface. Combined with the morphology of globular BioMnO_x_ wrapped in sheath, these findings suggest that BioMnO_x_ formation likely occurs on the cell surface of strain M125. This observation aligns with previously described mechanisms of microbial BioMnO_x_ production involving extracellular sheaths (Meng et al., 2009; Emerson and Ghiorse, 1992; Tebo et al., 2004; Corstjens et al., 1997). Apart from the wrapped globular BioMnO_x_, there were no bacterial cells in the sheath (Figure 5d and Figure 6a). That is likely due to the open-ended nature of immature sheaths, which may allow cell escape (Kunoh et al., 2020). These results indicate that the strain M125 is a sheath-producing bacterium, which is capable of enzymatic oxidation of Mn under neutral pH conditions. In natural environments, manganese oxides tend to accumulate along the external sheath. In addition, the morphology and size (150–350 nm) of these globular BioMnO_x_ produced by strain M125 resemble those formed via CueO enzyme mediated catalytic processes (Su et al., 2014). Furthermore, similar characteristics have also been reported for globular BioMnO_x_ produced in cocultures under anaerobic microbial Mn oxidation, where Mn_3_O_4_ and MnO_2_ were identified as primary components (Huang et al., 2023).

The structural evolution of BioMnO_x_ was investigated by comparing samples from 23-d and 90-d cultures. With extended cultivation, a portion of the BioMnO_x_ transitioned from an amorphous to a crystalline form (Figure 8), resulting in a mixture of amorphous (δ-MnO_2_) and crystalline states (Mn_3_O_4_). These findings indicated that there were various forms of manganese oxides in the BioMnO_x_ produced by strain M125. This conclusion was consistent with the results of LBB assay (Supplementary Figure S1a) and XPS analysis. The results confirmed the coexistence of oxidative products of Mn_3_O_4_ and MnO_2_ in the BioMnO_x_ (Figures 9a–c). Overall, the data confirm that BioMnO_x_ contains Mn in multiple oxidation states Mn(II), Mn(III), and Mn(IV) which can be explained by a two-step oxidation process mediated by strain M125: Mn(II) is first oxidized to Mn(III), then further to Mn(IV), leading to the formation of BioMnO_x_ composed of Mn_3_O_4_ (Mn_2_O_3_**·**MnO) and MnO_2_ phases. Similar multivalent manganese states in BioMnO_x_ have also been reported in previous studies (Wang et al., 2017; Learman et al., 2011b).

Previous studies have reported various structural forms of BioMnO_x_ produced by microorganisms. Some studies found predominantly amorphous structures, with porous δ-MnO_2_ (Wang et al., 2017; Meng et al., 2009; Zhou et al., 2015). While others observed distinct crystalline structures, including MnO_2_, Mn_3_O_4_, Mn_2_O_3_, or MnCO_3_ (Wang Y. et al., 2022; Hosseinkhani and Emtiazi, 2011). In this study, BioMnO_x_ generated by strain M125 showed a mixture of amorphous MnO_2_ and crystalline Mn_3_O_4_, presenting as two typical forms: nanosheets and globules. The BioMnO_x_ generated by strain M125 combines the benefits of both amorphous and crystalline structures, and different morphological forms. This unique characteristic may provide enhanced performance in terms of reactivity, stability, and applicability in different scenarios compared to the BioMnO_x_ reported in other studies. We believe that further exploration of the properties and applications of this strain and its BioMnO_x_ product will be valuable and may lead to new insights and advancements in the field.

This study also evaluated the Cd-adsorption capacity of BioMnO_x_. The BioMnO_x_ produced by strain M125 displayed a strong ability to adsorb cadmium (Figure 11), indicating its promising potential for practical applications. Thus, strain M125 could be a valuable candidate for producing microbial agents for cadmium removal. It should be noted that strain M125 and its BioMnO_x_ product exhibit excellent cadmium adsorption performance, which should be related to the unique structures of the BioMnO_x_. The observed nanosheets form of BioMnO_x_ in this study has an apparent loose and porous structure, these pores have good adsorption capacity. Besides, the BioMnO_x_ produced by strain M125 is not merely an inorganic manganese oxide but a complex organo-mineral composite incorporating manganese within an organic matrix, which also contributes to the adsorption of substances and the enrichment of ions.

In this study, a novel manganese-oxidizing bacterial strain, *Lysinibacillus xylanilyticus* M125, was isolated from polluted soil. And its Mn-oxidizing capacity was thoroughly investigated under laboratory conditions. Furthermore, detailed analyses were conducted on the structure, formation mechanism, and evolution of the BioMnO_x_ produced by this strain. Strain M125 showed good tolerance to varying pH levels and temperatures. BioMnO_x_ produced by strain also showed a strong cadmium adsorption capacity. This study provides a new and valuable bacterial resource, which can be used in the bioremediation of manganese and cadmium pollution.

## Data Availability

The 16S rRNA data presented in the study is deposited in the NCBI repository, accession number PV875934.
