# Supplementary material for: Isolation of a novel manganese-oxidizing bacterium Lysinibacillus xylanilyticus M125: characterization, structural evolution, and Cd-adsorption activity of biogenic Mn oxides produced by the strain
Source: Front Microbiol. 2025 Aug 21;16:1622784. doi: 10.3389/fmicb.2025.1622784 (PMC12410098; doi:10.3389/fmicb.2025.1622784)
Supplement: Supplementary file 1 [file Data_Sheet_1.docx]

Supplementary Material

**Isolation of a novel manganese oxidizing bacterium** ***Lysinibacillus xylanilyticus* M125: Characterization, structural evolution and Cd-adsorption activity of biogenic Mn oxides produced by the strain**

Xiaoju Li^1^, Xinyi Yuan^2^, Yuxia Wei^2^, Lianqi He^2^, Yuanyuan Li^2^, Meiquan Qiu^2^, Yang Liu^1^, Nannan Dong^1^, Chengjia Zhang^1^, Xin Pang^1,^^3,^^4^*

^1^State Key Laboratory of Microbial Technology, Shandong University, Qingdao 266237, China

^2^School of Life Sciences, Shandong University, Qingdao 266237, China

^3^Binzhou Institute of Technology, Weiqiao-UCAS Science and Technology Park, Binzhou 256606, China

^4^Gansu Institute of Shandong University, Lanzhou 730010, China

*** Correspondence:**

pangxin@sdu.edu.cn (Xin Pang)

† These authors contributed equally: Xiaoju Li, Xinyi Yuan

**Table S1.** Analysis and summary of isolated bacteria strains with manganese oxidation ability

| Strains | Current bacterial name | Is there pathogenicity | Possible bacterial species | LBB photos after 4 days of cultivation |
| --- | --- | --- | --- | --- |
| 1-F-2 | A-4 | be uncertain | *Bacillus paramycoides* | 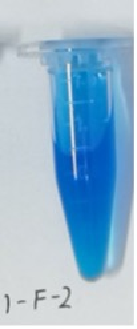 |
| 2-D | B-1 | Yes | *Acinetobacter tandoii* | 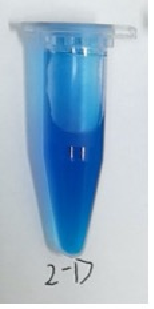 |
| 2-E | B-2 | Yes | *Acinetobacter haemolyticus* | 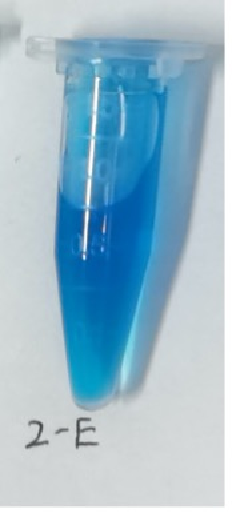 |
| 2-E-平 | B-3 | Yes | *Acinetobacter haemolyticus* | 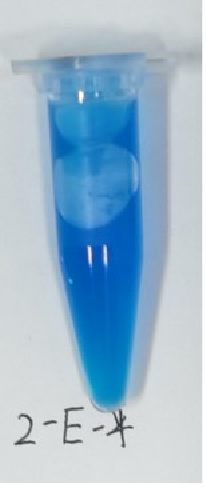 |
| 2-G | B-4 | Yes | *Acinetobacter tandoii* | 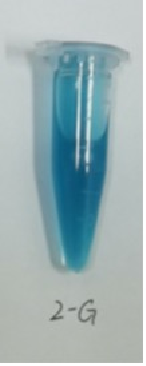 |
| 2-I-平-1 | B-5 | No | *Bacillus megaterium* | 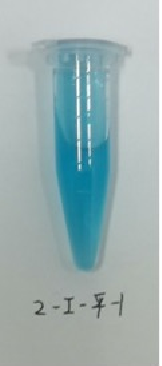 |
| 2-I-平-2 | B-6 | No | *Bacillus megaterium* | 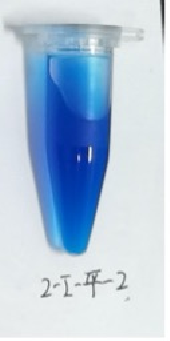 |
| 2.2.2a | B-7 | Yes | *Acinetobacter tandoii* | 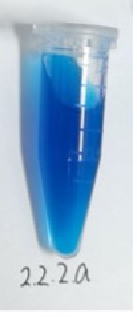 |
| 4-A-1 | C-1 | Yes | *Acinetobacter pittii* | 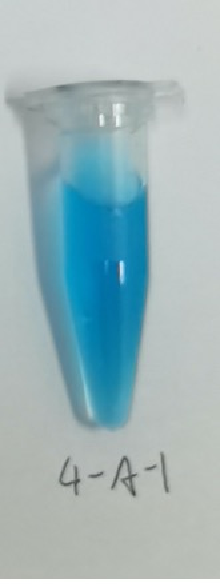 |
| 4-A-2-2 | C-3 | be uncertain | *Comamonas testosteroni* | 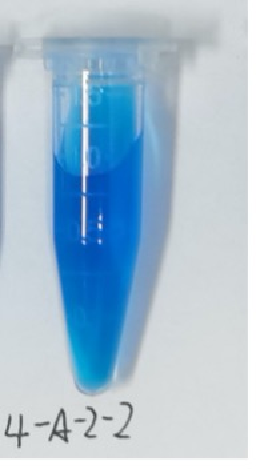 |
| 21JD-4-M-1 | D-2 | No | *Lysinibacillus pakistanensis* | 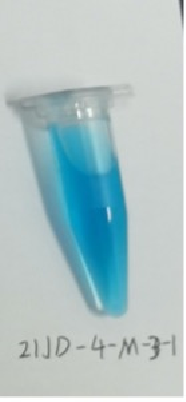 |
| **21JD-4-M-3-2** | D-4（M125）  **This study** | No | *Lysinibacillus xylanilyticus* | 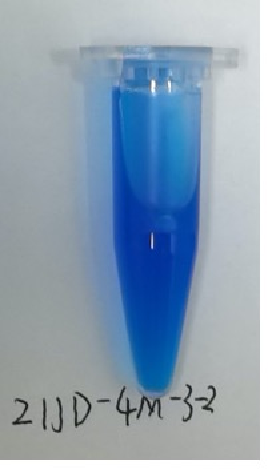 |

**Table S2.** 16S rRNA sequence of strain M125

| Strain | Sequence |
| --- | --- |
| M125 | TATACTGCAGTCGAGCGAACAGATAAGGAGCTTGCTCCTTTGACGTTAGCGGCGGACGGGTGAGTAACACGTGGGCAACCTACCTTATAGTTTGGGATAACTCCGGGAAACCGGGGCTAATACCGAATAATCTATTTCACTTCATGGTGAAATACTGAAAGACGGTTTCGGCTGTCGCTATAAGATGGGCCCGCGGCGCATTAGCTAGTTGGTGAGGTAACGGCTCACCAAGGCGACGATGCGTAGCCGACCTGAGAGGGTGATCGGCCACACTGGGACTGAGACACGGCCCAGACTCCTACGGGAGGCAGCAGTAGGGAATCTTCCACAATGGGCGAAAGCCTGATGGAGCAACGCCGCGTGAGTGAAGAAGGTTTTCGGATCGTAAAACTCTGTTGTAAGGGAAGAACAAGTACAGTAGTAACTGGCTGTACCTTGACGGTACCTTATTAGAAAGCCACGGCTAACTACGTGCCAGCAGCCGCGGTAATACGTAGGTGGCAAGCGTTGTCCGGAATTATTGGGCGTAAAGCGCGCGCAGGCGGTCCTTTAAGTCTGATGTGAAAGCCCACGGCTCAACCGTGGAGGGTCATTGGAAACTGGGGGACTTGAGTGCAGAAGAGGAAAGTGGAATTCCAAGTGTAGCGGTGAAATGCGTAGAGATTTGGAGGAACACCAGTGGCGAAGGCGACTTTCTGGTCTGTAACTGACGCTGAGGCGCGAAAGCGTGGGGAGCAAACAGGATTAGATACCCTGGTAGTCCACGCCGTAAACGATGAGTGCTAAGTGTTAGGGGGTTTCCGCCCCTTAGTGCTGCAGCTAACGCATTAAGCACTCCGCCTGGGGAGTACGGTCGCAAGACTGAAACTCAAAGGAATTGACGGGGGCCCGCACAAGCGGTGGAGCATGTGGTTTAATTCGAAGCAACGCGAAGAACCTTACCAGGTCTTGACATCCCGTTGACCACTGTAGAGATATGGTTTTCCCTTCGGGGACAACGGTGACAGGTGGTGCATGGTTGTCGTCAGCTCGTGTCGTGAGATGTTGGGTTAAGTCCCGCAACGAGCGCAACCCTTGATCTTAGTTGCCATCATTTAGTTGGGCACTCTAAGGTGACTGCCGGTGACAAACCGGAGGAAGGTGGGGATGACGTCAAATCATCATGCCCCTTATGACCTGGGCTACACACGTGCTACAATGGACGATACAAACGGTTGCCAACTCGCGAGAGGGAGCTAATCCGATAAAGTCGTTCTCAGTTCGGATTGTAGGCTGCAACTCGCCTACATGAAGCCGGAATCGCTAGTAATCGCGGATCAGCATGCCGCGGTGAATACGTTCCCGGGCCTTGTACACACCGCCCGTCACACCACGAGAGTTTGTAACACCCGAAGTCGGTGGGGTAACCTTTGGAGCCAGCCGCCGA |


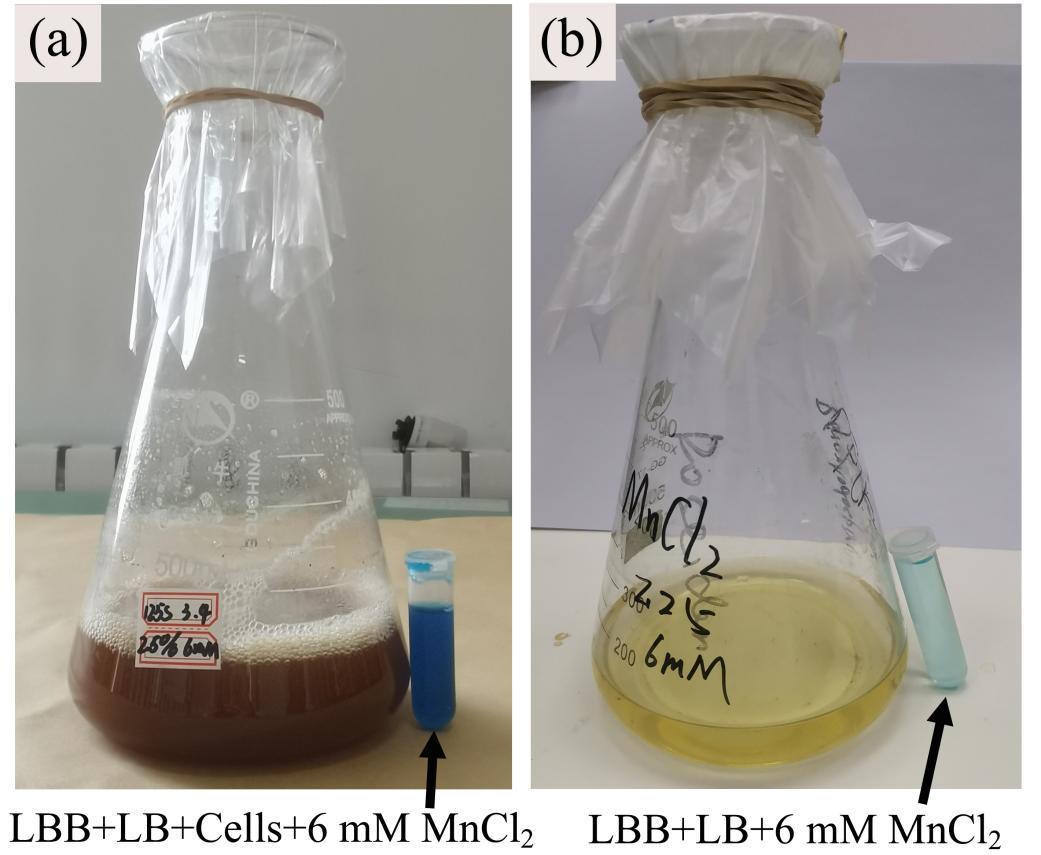


**Figure S1.** Liquid LB cultures containing Mn^2+^ (6 mM) with (a) and without (b) inoculation of strain M125. Culture conditions: temperature = 35 ℃, shaking = 180 r/min, initial pH = 7, and Mn^2+^ concentration 6 mM.


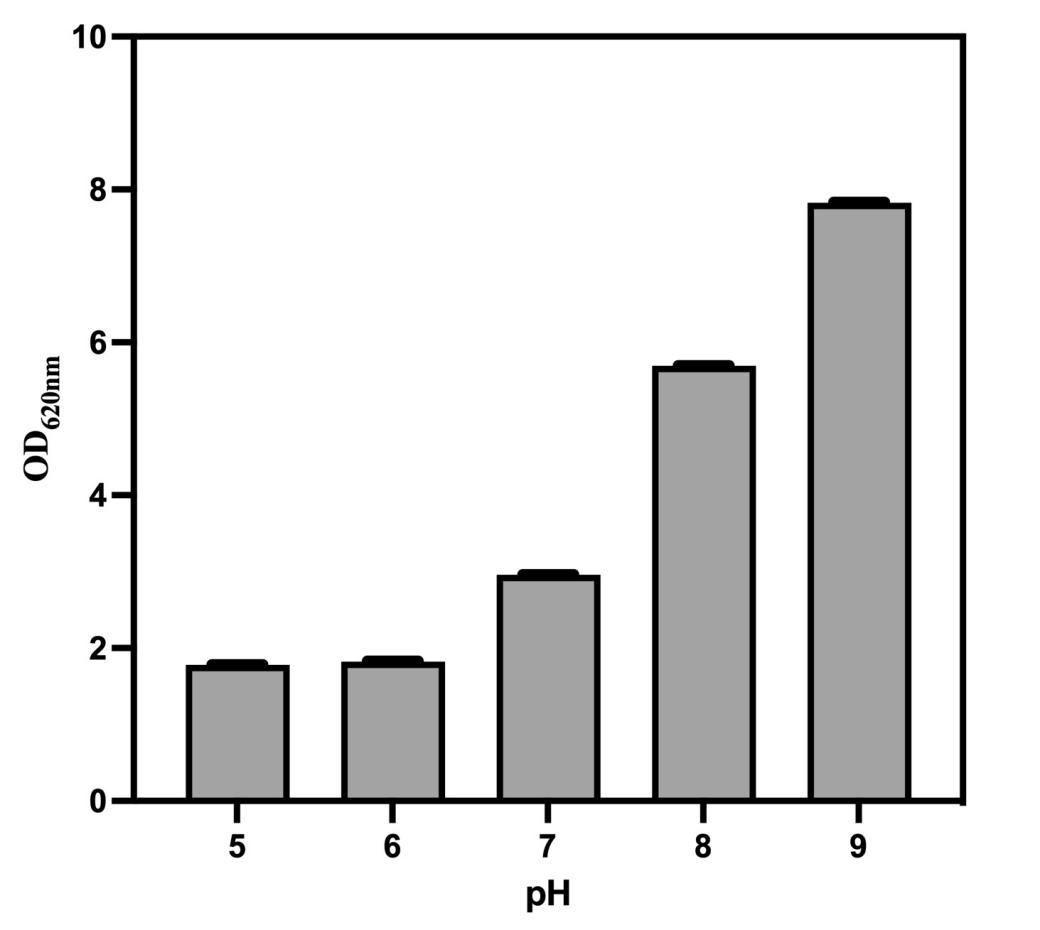


**Figure S2.** After cultivation for 8 d, OD _620nm_ values indicating the levels of Mn oxidation at different pH.


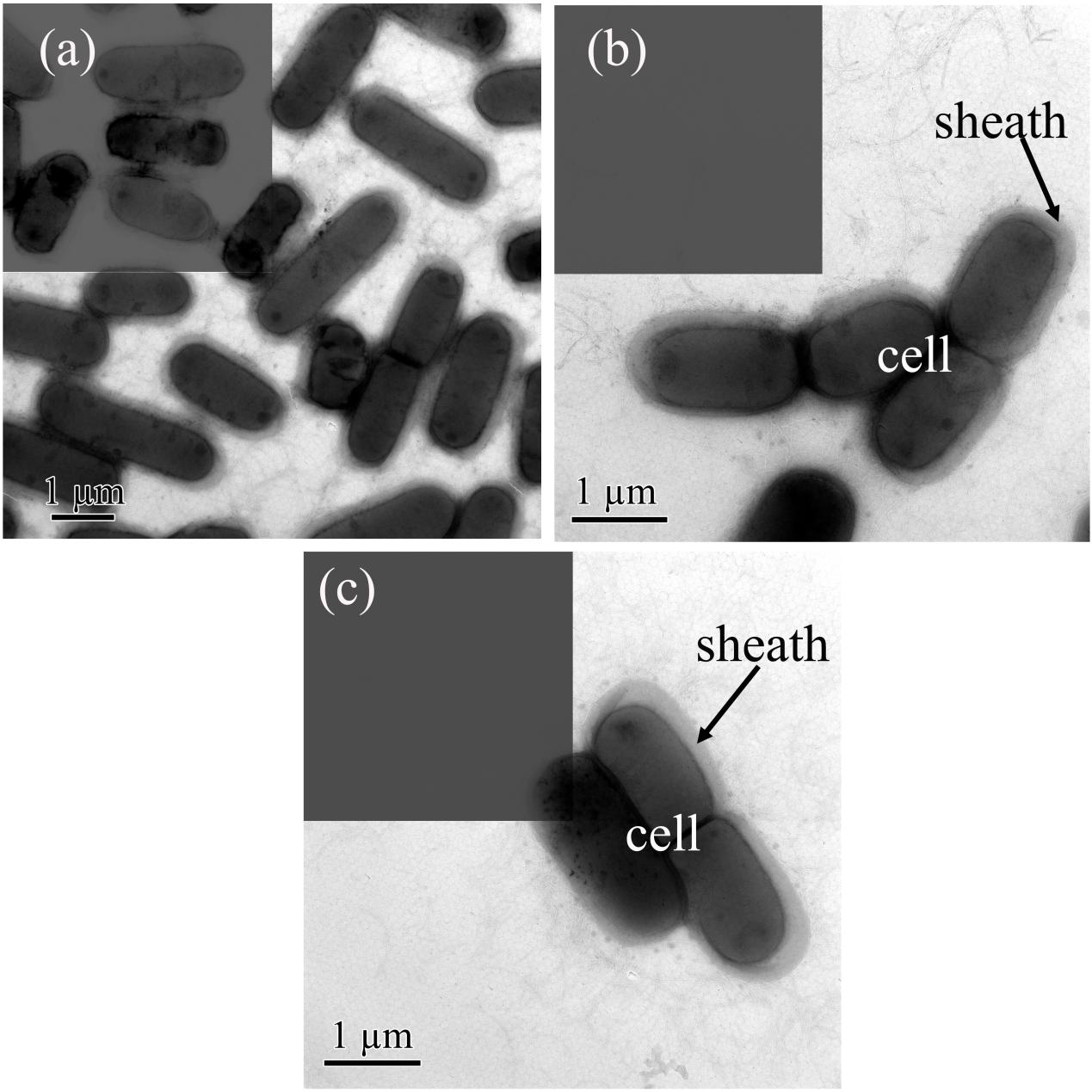


**Figure S3.** (a)-(c) TEM images showing the surface morphology of strain M125 cell wrapped by sheath morphology, showing that the cell surface is apparently wrapped by sheath.


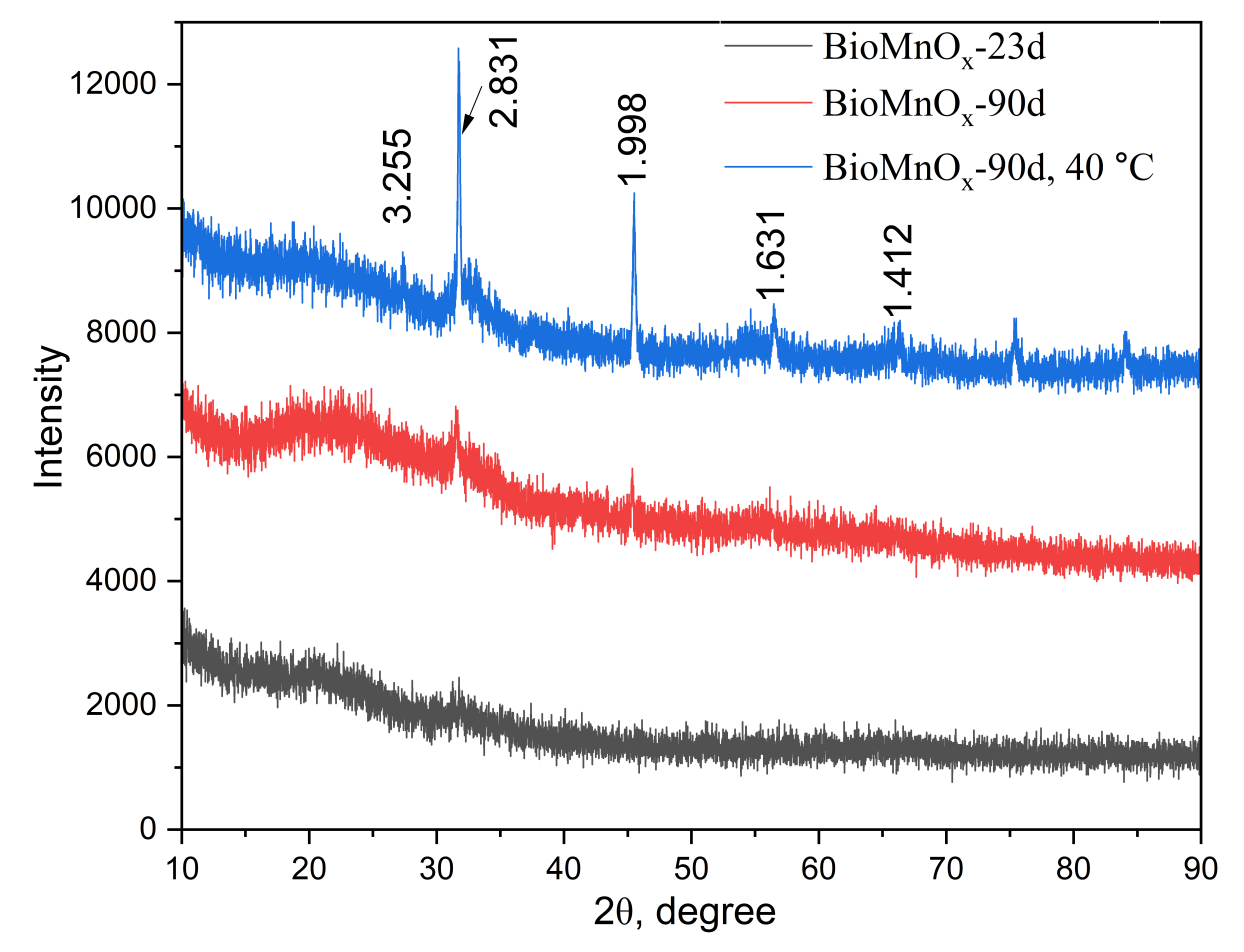


**Figure S4.** XRD patterns of BioMnO_x_ obtained under different culture conditions, showing an obvious structural evolution. At the initial, BioMnO_x_ is mainly amorphous. As the increment of cultivation time, especially under high temperature culture, BioMnO_x_ can transform into relatively good crystallization.

(b)

(a)


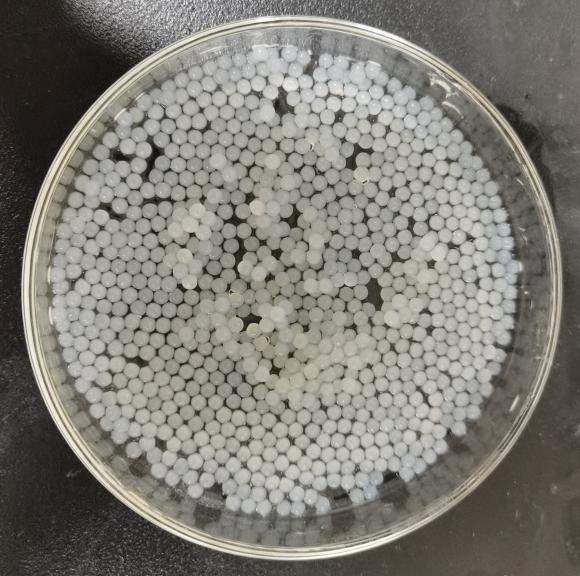

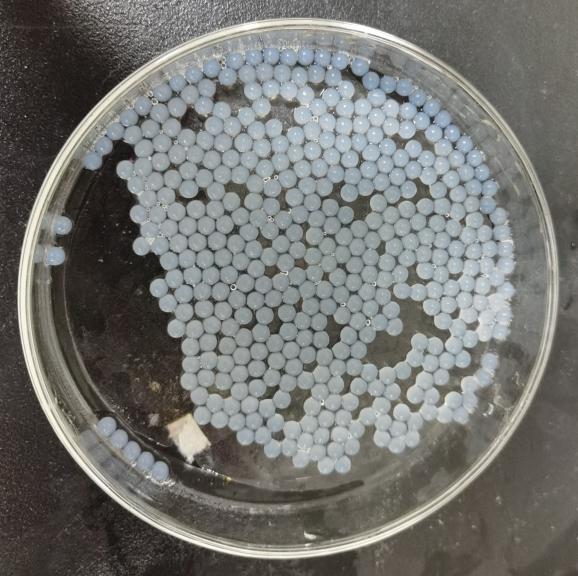


**Figure S5**. (a) 25% embedding of M125 in gel beads; (b) 35% embedding of strain M125 in gel beads.
